# Emerging Immunotherapy Targets in Early Drug Development

**DOI:** 10.3390/ijms26115394

**Published:** 2025-06-04

**Authors:** Daniel Morchón-Araujo, Greta Catani, Oriol Mirallas, Giulia Pretelli, Vicky Sánchez-Pérez, María Vieito, Irene Braña, Ricardo Pujol-Borrell, Elena Garralda, Alberto Hernando-Calvo

**Affiliations:** 1 Department of Medical Oncology, University Hospital of Salamanca, IBSAL, 37007 Salamanca, Spain; dmorchon@saludcastillayleon.es; 2 Vall d’Hebron Institute of Oncology, 08035 Barcelona, Spain; gcatani@alexanderfleming.org (G.C.); oriolmirallas@vhio.net (O.M.); giuliapretelli@vhio.net (G.P.); victoriasanchez@vhio.net (V.S.-P.); mvieito@vhio.net (M.V.); ibrana@vhio.net (I.B.); egarralda@vhio.net (E.G.); 3 Department of Medical Oncology, Alexander Fleming Institute, Buenos Aires 1426, Argentina; 4 Department of Medical Oncology, Vall d’Hebron Barcelona Hospital Campus, 08035 Barcelona, Spain; 5 Department of Cell Biology, Physiology and Immunology, Autonomous University of Barcelona, 08193 Bellaterra (Barcelona), Spain; ricardo.pujol@uab.cat; 6 Tumor Immunology and Immunotherapy Group, Vall d’Hebron Institute of Oncology, Vall d’Hebron Barcelona Hospital Campus, 08035 Barcelona, Spain

**Keywords:** immunotherapy, phase 1 trial, early drug development, checkpoint inhibitors, bispecific antibodies, tumor microenvironment

## Abstract

Immunotherapy has significantly changed the treatment paradigm for solid tumors, with immune checkpoint inhibitors now established in the management of many malignancies. Despite initial success, durable responses remain limited to a subset of patients, often less than 30%, due to both intrinsic and acquired resistance mechanisms. These challenges have prompted the development of next-generation immunotherapies. Recent efforts have expanded the scope of immunotherapy beyond PD-1/PD-L1 and CTLA-4 inhibition, focusing on new immune targets currently under investigation in early phase clinical trials. These include novel immune checkpoint inhibitors, immunomodulators targeting the tumor microenvironment, and bispecific antibodies. This review provides a comprehensive overview of emerging immune targets currently being investigated in early drug development, discussing their mechanisms of action, preliminary clinical outcomes, and potential future directions.

## 1. Introduction

**Figure 1:**
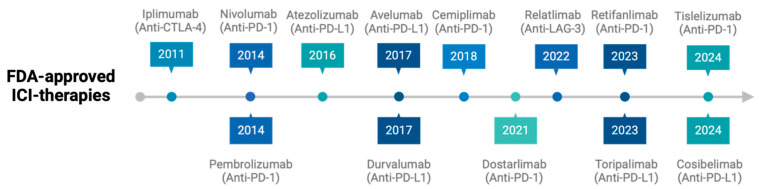
Timeline of FDA approvals for immune checkpoint inhibitors. Chronological representation of the first FDA approval for each immune checkpoint inhibitor. The timeline includes agents targeting PD-1, PD-L1, CTLA-4, and LAG-3, illustrating the sequential expansion of immunotherapy agents in oncology since 2011.

Immunotherapy has dramatically transformed the treatment landscape of solid tumors in recent years. Before the disruption in the clinic of immune checkpoint inhibitors (ICI), early immunotherapy efforts focused on harnessing the immune system using cytokines, such as interleukin-2 (IL-2) and interferon-alfa (IFN-α) in patients with melanoma and renal cancer [1,2,3,4]. Since the initial U.S. Food and Drug Administration (FDA) approval of ipilimumab in 2011 [5], which targets anti-cytotoxic T lymphocyte-associated antigen 4 (CTLA-4), followed by nivolumab and pembrolizumab in 2014, which target anti-programmed cell death protein 1 (PD-1) [6,7], there has been an exponential growth in the development of immunotherapy drugs. ICI have subsequently become a cornerstone in the management of several solid malignancies, either as monotherapy or in combination with chemotherapy agents, radiation therapy, or other targeted therapies. As of January 2025, there are 13 FDA-approved ICI for over 25 different tumor types and multiple indications (see Figure 1) [8].

**Figure 2:**
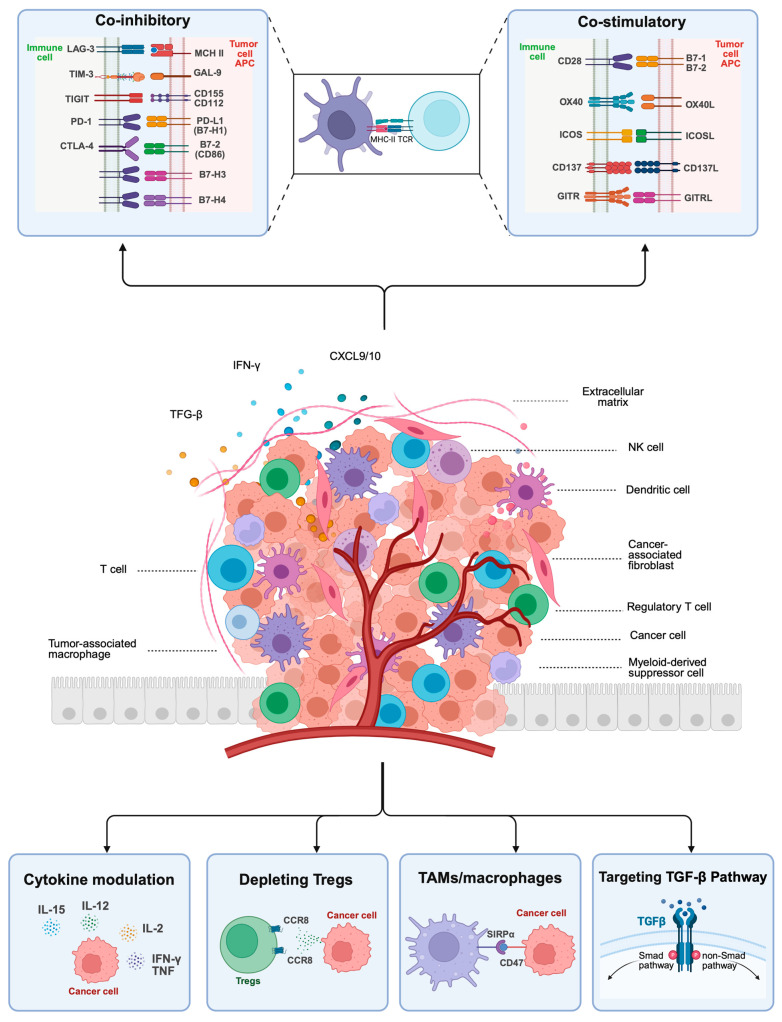
Key immune regulatory mechanisms and novel immunotherapeutic strategies in early phase clinical trials. The top of the figure shows the balance between coinhibitory and co-stimulatory molecules that modulate T-cell activation (see Section 2 and Section 3 of the main text). B7-H3 and B7-H4 have no known ligands. The central panel shows the tumor microenvironment (TME) as a dynamic network of tumor cells, immune cells, stromal components, and vasculature that collectively determine the immune response. At the bottom, four key approaches targeting the TME are presented: cytokine modulation (see Section 4), depletion of regulatory T cells, macrophage inhibition, and blocking TGF-β signaling (see Section 5).

Despite this initial success, only a subset of patients benefit long-term, and most tumors will ultimately develop acquired resistance mechanisms. Furthermore, in certain tumor types, response rates remain low or null, suggesting the presence of primary resistance mechanisms [9,10]. The wide range of efficacy rates observed across tumor types highlights the complex and dynamic interplay between tumor cells and the host immune system, as postulated in the immune editing hypothesis [11]. Intrinsic or primary resistance mechanisms include insufficient neoantigen load, loss of tumor antigen expression, and disruptions in the antigen presentation process [12]. Additionally, intrinsic alterations in tumor signaling pathways (e.g., MAPK, WNT/β-catenin, and PI3K) can enable immune evasion through immunosuppressive cytokine secretion, T-cell exclusion, and impaired antigen presentation, thereby rendering ICI ineffective [9,13]. Tumor cells can also develop resistance by downregulating antigen expression or disrupting interferon-gamma (IFN-γ) signaling pathways [14]. Constitutive expression of immunosuppressive surface ligands (e.g., programmed death-ligand 1 [PD-L1]) [15] or upregulation of alternative immune checkpoints (e.g., lymphocyte activation gene [LAG-3], T-cell membrane protein [TIM-3], and T-cell immunoreceptor with immunoglobulin and ITIM domains [TIGIT]) can further limit antitumor activity [14,16]. Beyond these intrinsic mechanisms, the tumor microenvironment (TME) plays a critical role in immune evasion (see Figure 2). Tumors may recruit immunosuppressive cells, including regulatory T cells (Tregs), myeloid-derived suppressor cells (MDSCs), and tumor-associated macrophages (TAMs), all of which suppress effector T-cell activity [17,18,19]. Additionally, immunosuppressive cytokines, particularly transforming growth factor-beta (TGF-β), have been shown to contribute to resistance to immunotherapy [20]. Metabolic changes within the TME may also support a hostile environment for immune cells, thereby promoting tumor survival and immune evasion.

Advances in our understanding of the complex interplay between cancer cells, the TME, and the host immune system have driven the identification of novel therapeutic targets. In this context, early drug development, particularly phase I clinical trials, plays an essential role in oncology by enabling the initial assessment of investigational drug safety, tolerability, and early efficacy signals. Over the past decade, there has been a marked increase in the number of immunotherapy phase I clinical trials, primarily using monoclonal antibodies (mAbs) targeting the PD-1/PD-L1 axis and their combinations [21]. However, recent trends show a shift in focus towards bispecific antibodies (bsAbs) and immune cell engagers to overcome resistance and improve activity in cold or non-immunogenic tumor types [22]. This evolution underscores the rapidly changing landscape of immunotherapy drug development.

This review aims to provide a comprehensive overview of novel immune targets currently under investigation in early drug development, including alternative checkpoints, co-stimulatory receptors, cytokine-based agents, and modulators of myeloid and stromal components of the TME. To ensure a concise scope, cell therapies and vaccines have been excluded. We highlight the mechanisms of action, clinical progress, and potential future directions to address current limitations in the field of cancer immunotherapy.

## 2. Immune Checkpoint Inhibitors Beyond PD-1/PD-L1 and CTLA-4

Adaptive immune responses driven by T cells are fundamental for antitumor immunity. T-cell activation is regulated by a balance between co-stimulatory and inhibitory signals [23]. While inhibitory checkpoints play a key role in maintaining immune tolerance [24], persistent antigen stimulation can lead to T-cell exhaustion and impaired effector function [25]. The immune checkpoint blockade has proven to be a remarkably efficacious strategy in recent years. However, our growing understanding of resistance to first-generation ICI has driven the search for alternative immune checkpoints as novel therapeutic targets [16].

### 2.1. Lymphocyte Activation Gene 3 (LAG-3 and CD223)

LAG-3 is a transmembrane protein belonging to the immunoglobulin (Ig) superfamily. Initially identified in 1990 due to its structural similarity to the CD4 receptor [26], this inhibitory receptor was primarily studied in activated T cells. However, it is also expressed on Tregs, natural killer (NK) cells, dendritic cells (DCs), and even B cells [27]. Its primary postulated ligand is major histocompatibility complex (MHC) class II [28], but additional ligands have been identified, including galectin-3, LSECtin, FGL1, T-cell receptor (TCR)-CD3 complex [27], and FCRL6 [29]. These interactions modulate LAG-3-mediated immune suppression, although their precise roles remain incompletely understood. The interaction of LAG-3 with MHC-II and its other ligands inhibits the proliferation, activation, and cytokine secretion of CD4^+^ and CD8^+^ T cells, thereby contributing to maintaining immune tolerance [28]. Indeed, LAG-3 co-expression with PD-1 is recognized as a hallmark of “T-cell exhaustion” [30]. Preclinical models have shown greater efficacy with the dual blockade of PD-1 and LAG-3, resulting in a synergistic effect and enhanced antitumor activity [31]. Thereby, the LAG-3 blockade has emerged as a promising therapeutic strategy in cancer immunotherapy.

In recent years, different approaches have been explored in early phase clinical trials, including LAG-3 antagonist mAbs, Ig-fusion proteins, or bsAbs. Notably, Opdualag, a fixed-dose combination of relatlimab-rmbw (anti-LAG-3, human IgG4 mAb) and nivolumab (anti-PD-1, human IgG4 mAb), has become the first “new-generation checkpoint inhibitor” to receive FDA approval. The RELATIVITY-047 phase II/III clinical trial evaluated Opdualag versus nivolumab in treatment-naïve unresectable or metastatic melanoma. The study reported a median overall survival (mOS) of 51 months with the combination therapy, compared to 34.1 months in the nivolumab monotherapy arm after a median follow-up of 33.8 months [32]. However, this clinical activity was associated with a higher incidence of toxicity, with grade 3–4 (G3/4) treatment-related adverse events (TRAEs) occurring in 22% of patients in the Opdualag arm, compared to 12% observed in the nivolumab monotherapy group [32].

The success of this combination in melanoma likely reflects the significant role of LAG-3 as a coinhibitory receptor contributing to T-cell exhaustion in a substantial proportion of these patients. However, the subsequent failure of the same combination to demonstrate meaningful clinical activity in gastric and colorectal cancers underscores the crucial role of tumor-specific molecular context and immune landscapes [33,34]. A phase III trial on first-line non-small cell lung cancer (NSCLC) is ongoing (NCT06561386). Favezelimab, a different anti-LAG-3 mAb, was tested in combination with pembrolizumab and discontinued after failing in a phase III trial in microsatellite-stable (MSS) colorectal cancer (CRC) [35]. Other anti-PD-1 plus anti-LAG-3 combinations are still under evaluation (see Table 1). These mixed results suggest that LAG-3 may not be a universally dominant resistance mechanism across all tumor types. For instance, gastrointestinal cancers might be characterized by different immunosuppressive pathways (e.g., MDSCs, alternative checkpoint expression, or distinct stromal barriers), lower baseline T-cell infiltration, or a less critical functional reliance on the LAG-3/MHC-II axis compared to melanoma. Furthermore, the specific patient populations within these trials (e.g., unselected biomarker or prior treatment exposure) could have contributed to the lack of observed efficacy.

The exploration of triplet combinations (e.g., PD-1, LAG-3, and TIM-3 [NCT04370704, NCT03744468] or bsAbs simultaneously targeting PD-1 and LAG-3 receptors represents a rational approach to address the complexity of T-cell exhaustion, where multiple inhibitory receptors are often upregulated [36]. These strategies aim for a more profound reversal of T-cell dysfunction, although they will also require careful management of potential overlapping toxicities.

Alternative approaches beyond antagonistic mAbs have also emerged, offering a distinct mechanistic approach. Eftilagimod alpha, a first-in-class soluble LAG-3 protein which acts as MHC class II agonist, has recently shown an overall response rate (ORR) of 55% and a disease control rate of 87.5% in combination with anti-PD-1 and chemotherapy in advanced NSCLC (phase I INSIGHT-003 trial [NCT03252938]) [37]. Its agonistic binding to MHC class II aims to broadly activate antigen-presenting cells (APCs), thereby boosting T-cell priming and immune responses. Conversely, the more modest ORR of 8.3% observed in the TACTI-002 phase II study in second-line NSCLC (eftilagimod alpha plus pembrolizumab) highlights the influence of treatment setting, patient population, and combination partners. This trial reported a median progression-free survival (mPFS) of 2.1 months and a mOS of 9.9 months. Of note, 82% had a PD-L1 tumor proportion score (TPS) <50%, and 67% had received a chemotherapy doublet plus pembrolizumab as the previous line, representing a more challenging and likely immune-exhausted population [38]. The phase III, TACTI-004 (NCT06726265), is ongoing to evaluate eftilagimod alpha in combination with pembrolizumab and chemotherapy as a first-line treatment for patients with advanced NSCLC, and data will be forthcoming.

However, significant knowledge gaps remain concerning the underlying molecular mechanisms that regulate LAG-3 function, the biological relevance of its different ligands in specific contexts, and the identification of biomarkers that would allow for selecting patients who would benefit most from therapies directed against this checkpoint. For instance, a numerically higher progression-free survival (PFS) was observed in the phase III registrational study of relatlimab in combination with nivolumab for those tumor samples with LAG-3 overexpression assessed by immunohistochemistry [39]. Additionally, results from early phase clinical trials suggest that LAG-3 overexpression may be associated with increased activity for this treatment combination [40,41]. Addressing these gaps is essential for optimizing patient selection, designing more effective combination therapies, and overcoming resistance to LAG-3 targeted treatments.

### 2.2. T-Cell Membrane Protein 3 (TIM-3)

TIM-3 is a transmembrane protein first identified in 2002 [42]. Originally identified on T-helper 1 cells, TIM-3 is currently known to be expressed by various immune cells, including CD4^+^ and CD8^+^ T cells, Tregs, NK cells, and other myeloid cells [43]. TIM-3 interacts with multiple ligands, including galectin-9, phosphatidylserine, CEACAM1, and HMGB1, modulating immune signaling pathways to dampen effector immune responses and contributing to an immunosuppressive TME [44]. TIM-3 is also upregulated on exhausted T cells. Interestingly, in preclinical models, TIM-3^+^/PD-1^+^ tumor-infiltrating lymphocytes (TILs) are characterized by significantly impaired proliferation and reduced cytokine production (IL-2, tumor necrosis factor [TNF], and IFN-γ) with a more severe exhausted phenotype than TIM-3^−^/PD-1^+^ TILs [45]. Furthermore, increased TIM-3 expression on TILs has been observed in tumors progressing following their response to an anti-PD-1 blockade [16]. TIM-3 expression has also been correlated with worse outcomes in several tumors [46]. Considering all these factors, TIM-3 has become a promising target for next-generation ICI, primarily explored in combination strategies (see Table 1).

Early clinical investigations of TIM-3 targeting mAbs have predominantly focused on combinations with PD-1/PD-L1 inhibitors, a strategy underpinned by the co-expression of these receptors on severely exhausted T cells, especially in tumor resistance prior to a checkpoint blockade. However, these initial efforts have yielded varied and often modest clinical outcomes alongside differing safety profiles.

For instance, sabatolimab (MBG453), a humanized IgG4 mAb, when combined with the anti-PD-1 mAb spartalizumab in a phase I study (NCT02608268), demonstrated a partial response rate of only 6% across a mixed cohort of advanced solid tumors, including CRC, NSCLC, malignant perianal melanoma, and small-cell lung cancer (SCLC) [47]. A significant challenge was highlighted in the phase II part of this study, where no objective responses were observed in melanoma and NSCLC patients who had previously progressed on PD-1/PD-L1 therapy [48]. This lack of efficacy in a refractory setting was accompanied by a notable toxicity burden, with 46% of patients experiencing G3/4 TRAEs [48]. Similarly, the anti-TIM-3 mAb LY3321367 (NCT03099109), evaluated both as monotherapy and in combination with the anti-PD-L1 mAb, LY3300054, also showed limited antitumor activity in patients with relapsed or refractory advanced solid tumors. While the phase I study reported an acceptable safety profile and favorable pharmacokinetics (PK)/pharmacodynamics (PD), the ORR in the combination expansion cohorts was 4% [49].

In contrast to these findings, the AMBER phase I trial investigating cobolimab (TSR-022) in combination with PD-1 inhibitors (dostarlimab or nivolumab) offered more encouraging preliminary signals. This combination was reported as well tolerated and achieved responses in 19% of patients with advanced solid tumors [50]. While direct cross-trial comparisons are confounded by differences in patient populations, prior therapies and specific PD-1 partners, the higher ORR observed with cobolimab warrants further investigation. A detailed comparative analysis of the specific types and frequencies of G3/4 TRAEs between sabatolimab, LY3321367, and cobolimab combinations would be crucial for understanding the therapeutic index of different TIM-3 antibodies and their respective combination regimens.

The combinatorial strategy is further evolving with trials like COSTAR Lung (NCT04655976). This phase II/III study is assessing cobolimab in combination with dostarlimab and docetaxel as a second-line treatment for advanced NSCLC following disease progression after anti-PD-1/PD-L1 therapies. This triplet combination represents a shift towards integrating chemotherapy, potentially for its immunomodulatory effects or to sensitize tumors to a dual checkpoint blockade.

In summary, while the biological rationale for targeting TIM-3 remains compelling, particularly for reversing T-cell exhaustion in the TME, early clinical data have shown that translation into robust clinical efficacy is challenging. The advent of bsAbs that target TIM-3 and other immune checkpoints could enhance efficacy by simultaneously modulating multiple immunosuppressive pathways. Addressing the current limitations through integrated preclinical and clinical studies, focusing on patient selection biomarkers and optimized combination approaches, is essential for fully leveraging the potential of TIM-3 targeted therapies.

### 2.3. T-Cell Immunoreceptor with Ig and ITIM Domains (TIGIT)

The T-cell immunoreceptor with Ig and ITIM domains (TIGIT) was first described in 2009 [51]. This inhibitory receptor is expressed on CD4^+^ and CD8^+^ T cells, NK cells, and Tregs. From a structural point of view, TIGIT is a transmembrane glycoprotein of the Ig superfamily and is part of a complex pathway involving other inhibitory (e.g., CD96 and CD112R) and co-stimulatory (CD226) receptors that compete for the same ligands [52]. In particular, two main ligands have been identified for TIGIT: CD112 (Nectin-2) and CD155 (poliovirus receptor, PVR), which are broadly expressed on antigen-presenting cells (APCs) and tumor cells [52]. TIGIT suppresses antitumor immunity through multiple mechanisms, including intrinsic inhibition of effector T cells and NK cells, blocking CD226 co-stimulation, enhancing Tregs function, and promoting immunosuppressive DCs, which increase IL-10 secretion [53,54,55]. TIGIT is typically co-expressed with other exhaustion markers, such as PD-1, LAG-3, or TIM-3, suggesting a potential resistance mechanism to first-generation ICI [36]. In this regard, simultaneous inhibition of multiple targets using customized treatment combinations may be an effective strategy to reinvigorate exhausted T cells. TIGIT expression has also been described as a poor prognostic marker in various tumor types, such as NSCLC or melanoma [56]. In preclinical models, the dual PD-1/TIGIT blockade has been shown to provide the additive expansion and functional activity of tumor antigen-specific CD8^+^ T cells and TILs [54].

Multiple innovative TIGIT-targeting agents have entered clinical development (see Table 1). Tiragolumab, a first-in-class, fully human IgG1/kappa anti-TIGIT mAb with an intact Fc region, demonstrated safety and preliminary efficacy in the phase Ia/Ib GO30103 trial (NCT02794571), both as monotherapy and with atezolizumab [57]. This was bolstered by the phase II CITYSCAPE (NCT03563716) in chemotherapy-naïve, PD-L1-positive NSCLC, where a combination of tiragolumab plus atezolizumab significantly improved ORR (31.3% vs. 16.2%, *p* = 0.031) compared to atezolizumab with a comparable safety profile (G3/4 TRAEs: 21% vs. 18%), suggesting good tolerability for the dual blockade [58]. However, subsequent large, randomized phase III trials, SKYSCRAPER-01 and SKYSCRAPER-02, failed to meet their primary endpoints of PFS and OS as recently disclaimed [59]. Similarly, phase II trials investigating vibostolimab, another anti-TIGIT mAb, in combination with pembrolizumab (KeyVibe-003 in NSCLC and KeyVibe-007 in SCLC) also failed to demonstrate significant clinical benefit [60]. These failures led to critical evaluation regarding optimal TIGIT engagement, the contribution of FC-mediated effector functions, and patient selection strategies.

In this context, the development path of domvanalimab (AB154) offers a potentially differentiating perspective. Domvanalimab is an Fc-silent anti-TIGIT mAb designed to avoid antibody-dependent cellular cytotoxicity (ADCC) of TIGIT-expressing effector T cells. Encouragingly, the phase II (ARC-10) trial reported that domvanilimab combined with the anti-PD-1 mAb zimberelimab improved PFS and OS compared to zimberelimab monotherapy or chemotherapy in PD-L1-high advanced NSCLC [61]. While direct comparison of toxicity across programs is challenging without standardized reporting, the Fc-silent nature of domvanalimab may contribute to a distinct safety and efficacy profile compared to Fc-component mAbs like tiragolumab, although this requires further evaluation. Domvanalimab is currently being investigated in several phase III clinical trials: in combination with zimberelimab and chemotherapy for upper gastrointestinal tumors (STAR-221 trial, NCT05568095); with chemotherapy in untreated advanced NSCLC (STAR-121, NCT05502237); and with the anti-PD-L1 durvalumab following concurrent chemoradiotherapy in stage III unresectable NSCLC (PACIFIC-8, NCT05211895), exploring its utility in a curative-intent setting. Another agent, ociperlimab, is also being evaluated in combination with the anti-PD-1 tislelizumab in the phase III ADVANTIG-302 trial (NCT04746924) for advanced NSCLC. The field is further evolving with the investigation of bsAbs, which simultaneously target TIGIT and PD-1 (e.g., AZD2936, NCT04995523; BC008-1A, NCT06773507) or TIGIT and PD-L1 (e.g., HLX301, NCT05102214), aiming to maximize pathway inhibition within the TME.

In summary, the TIGIT pathway remains a target of high interest, but its clinical translation has proved more complex. Early positive signals from phase II studies, such as CITYSCAPE (tiragolumab), were not consistently replicated in larger phase III trials for some Fc-competent antibodies. The varied efficacy rates highlight the need to better understand TIGIT biology, the impact of antibody engineering (e.g., Fc competent vs. Fc-silent like domvanalimab), appropriate patient selection (beyond PD-L1 status), and optimal combination partners. The comparable G3/4 TRAE rates seen in comparison to anti-PD-1/PD-L1 as monotherapy suggested that the dual TIGIT/PD-1 blockade could be well tolerated, but comprehensive safety comparisons are still emerging. Ongoing phase III trials, along with the development of bsAbs, will be critical in defining the future therapeutic role of TIGIT in cancer.

### 2.4. Other Inhibitory Checkpoints

The B7 family comprises a group of structurally related transmembrane proteins within the Ig superfamily. This family has transformed cancer therapy through the advent of ICI targeting PD-1, its ligand PD-L1 (B7-H1), and CTLA-4. In addition to these well-established therapeutic targets, ongoing research is increasingly directed toward less-explored members of the B7 family. Among these, B7-H3 (CD276), B7-H4 (VTCN1), and B7-H5 (VISTA/PD-1H) present distinct expression profiles, mechanisms of action, and therapeutic strategies, collectively offering new avenues to overcome resistance and enhance antitumor immunity.

B7-H3 and B7-H4 share several features that make them attractive targets for novel cancer therapies, most notably their frequent overexpression on diverse tumor types coupled with limited expression in healthy tissues. This differential expression provides a theoretical therapeutic window, particularly for modalities such as antibody-drug conjugates (ADCs) and bsAbs. Both are primarily recognized for their immune-inhibitory functions, contributing to an immunosuppressive TME [62,63,64].

B7-H3 is particularly widespread across several cancer types, where its presence is often correlated with poor prognosis [65,66]. While now predominantly viewed as an immune checkpoint-suppressing T-cell activity, B7-H3 also promotes direct pro-tumoral effects through various oncogenic signaling pathways. The lack of a definitively identified physiological ligand remains a challenge for its complete biological characterization. Clinically, its role as a tumor-associated antigen (TAA) has led to the development of chimeric antigen receptor T (CAR-T) cells, ADCs, and bsAbs, which are currently being evaluated in early phase clinical trials [67,68]. The Fc-optimized mAb enoblituzumab initially demonstrated encouraging ORRs of 33.3% and 35.7% in patients with HNSCC and NSCLC, respectively, when combined with pembrolizumab, albeit with notable G3/4 TRAEs (28.6%) [69]. However, a significant setback occurred when a phase II-assessing enoblituzumab in combination with retifanlimab (anti-PD-1 mAb) or tebotelimab, a bsAb PD-1 × LAG-3 antibody, was discontinued due to a high rate of fatal hemorrhagic events in both treatment arms (11.3%), underscoring critical safety considerations for specific B7-H3-targeted agents and combinations [70]. Despite this, B7-H3 remains a highly pursued target, largely due to its prevalent overexpression and the advancement of multiple ADCs, with ongoing investigations in diverse settings.

B7-H4 shares with B7-H3 a predominantly tumor-restricted expression profile, particularly in cancers such as breast, ovarian, and endometrial cancers, with minimal normal tissue presence [71]. It contributes to immune evasion, and its expression has been linked to reduced immune cell infiltration in the TME [63,64]. Early data for the mAb alsevalimab suggested a favorable safety profile with the most common grade 1–2 TRAEs (16.7% diarrhea and fatigue) [72], but current clinical development for B7-H4 strongly emphasizes ADCs (e.g., NCT05377996, NCT06336707, and NCT06774963) and bsAbs like ABL103 (B7-H4 × 4-1BB; NCT06126666) or GEN1047 (B7-H4 × CD3; NCT05180474). Compared to B7-H3, B7-4 targeting is at an earlier stage of clinical validation. Its promise largely depends on demonstrating potent and selective antitumor activity through these ongoing trials.

B7-H5, also known as V-domain Ig suppressor of T-cell activation (VISTA), possesses unique mechanistic features that distinguish it within the B7 family [73,74]. It is constitutively expressed at high levels in myeloid-derived immune cells (monocytes, macrophages, granulocytes, and DCs, as well as in subsets of T cells) [75,76]. Unlike other immune checkpoints that are upregulated in response to activation, VISTA is broadly expressed at a steady state [77]. Notably, its expression is implicated in resistance to existing anti-PD-1/PD-L1 and anti-CTLA-4 therapies, suggesting its role as a compensatory immune evasion mechanism [78].

Clinically, VISTA’s role in innate and adaptive immunity, especially regarding ICI resistance, makes it a highly promising, albeit complex, target, with development focused on combination strategies with PD-1 inhibitors (see Table 1). To mitigate potential toxicities from its broad expression, innovative antibody engineering is prominent, exemplified by agents designed for minimal Fc effector function (e.g., HMBD-002, an IgG4 mAb) or pH-selective activation within the acidic TME (e.g., SNS-101), which are both in phase I trials (NCT05082610; NCT05864144) [79,80]. Beyond antibodies, the oral small-molecule CA-170 dually antagonizes VISTA and PD-L1, providing a distinct approach, though robust efficacy data are needed [81]. VISTA’s therapeutic promise is based on its potential to reprogram the suppressive myeloid landscape and resensitize tumors to ICI.

## 3. Targeting Co-Stimulatory Pathways

While T cells recognize tumor antigens presented by APCs via their TCRs, this signal alone may be insufficient to generate a sustained antitumor immune response. Co-stimulatory signals are crucial to enhance T-cell activation, proliferation, and survival. While CD28 serves as one of the first steps, additional signals are required to support expansion and prevent T-cell exhaustion. Co-stimulatory receptors can be categorized into two big families. The TNF receptor superfamily (TNFRSF) includes the glucocorticoid-induced TNF receptor family-related protein (GITR), OX40, and 4-1BB, and the Ig superfamily, which comprises receptors such as CD28 and the inducible T-cell co-stimulatory (ICOS).

Antibodies have been designed to activate co-stimulatory receptors by mimicking natural ligand binding, but the development of agonist drugs has been more complex. Unlike antagonist antibodies, which primarily aim to block receptors competitively, agonist antibodies rely on multiple factors, including their interaction with Fcγ receptors (FcγRs). Importantly, the nature of this FcγR engagement can influence outcomes within the TME [82]. Binding to inhibitory FcγRs can amplify agonist signaling through antibody-crosslinking, whereas engagement of activating FcγRs on NK cells or macrophages can drive Treg depletion through ADCC or antibody-dependent cell-mediated phagocytosis (ADCP) [82]. This dual role highlights the nuanced design required for effective agonist-antibody therapies. Targeting these co-stimulatory receptors may have a synergistic effect to reinvigorate T-cell functioning in combination with anti-PD-1 therapy and improve overall therapeutic outcomes [83]. Such combinations may more effectively reinvigorate exhausted T cells within the TME, enhance the magnitude and quality of the antitumor T-cell response, improve the generation of durable T-cell memory, and potentially overcome resistance to ICI monotherapy.

### 3.1. OX40

OX40 is a type 1 transmembrane glycoprotein primarily expressed by activated T cells. Its expression is only induced following antigen recognition, making OX40 a highly specific marker of activated T cells, particularly effector and memory T cells, as well as certain Tregs [84]. OX40 interacts with its ligand, OX40L (CD134L), which is predominantly expressed on APCs [84]. This interaction promotes T-cell survival and proliferation and enhances memory T-cell formation [85]. OX40 is primarily expressed on TILs in various cancers, and its prognostic value has been controversial, with either favorable or unfavorable outcomes depending on the specific T-cell subset [86,87,88]. OX40-selective expression on activated T cells makes it an attractive target for immunotherapy, as it spares naïve T cells, potentially reducing the risk of immune-related adverse effects.

Several OX40-targeting agents have been developed, each with distinct PK and PD properties. For instance, INCAGN01949, an IgG1-agonist mAb, was designed to facilitate ADCC, thereby depleting Tregs within the TME [89], while ivuxolimab, an IgG2 mAb, aimed to avoid such ADCC-mediated effects [90]. Despite these biological advancements, OX40-agonist mAbs have demonstrated limited clinical efficacy as monotherapy in solid tumors, with suboptimal response rates reported [89,90,91]. Consequently, several early OX40 agonists (e.g., revdofilimab, ivuxolimab, INCAGN01949, and MEDI0562) have been withdrawn from development.

However, novel OX40 agonism strategies have emerged. INBRX-106, a hexavalent OX40 agonist designed for enhanced receptor clustering, has generated renewed interest and is currently being investigated in combination with pembrolizumab in a phase II/III trial for the first-line treatment of advanced HNSCC with a PD-L1-combined positive score (CPS) > 20 [92]. Other novel agonists, such as BGB-A445, HFB301001 [93], and HLX51 (see Table 2), are being investigated in early phase trials. Notably, GEN1055, which employs Genmab’s proprietary HexaBody^®^ technology, has recently entered clinical development (NCT06391775). This approach enables clustering of OX40 receptors independently of FcγR-mediated crosslinking. BsAbs, such as FS120 (OX40 × 4-1BB), and EMB-09 (OX40 × PD-1) also offer alternative strategies, although clinical efficacy data remain pending. Collectively, these ongoing efforts highlight the continued pursuit of optimizing OX40-targeted therapies.

### 3.2. 4-1BB (CD137)

4-1BB, also known as CD137, was identified in the 1980s [94]. However, it is in recent years that 4-1BB has been established as a main focus in immuno-oncology. 4-1BB is an inducible transmembrane glycoprotein primarily expressed on activated immune cells, such as CD8^+^ and CD4^+^ T cells, NK cells, and DCs [95,96]. The biological functions of 4-1BB include the activation of T cells and function by amplifying clonal expansion and effector responses [97]. Beyond T cells, 4-1BB signaling influences NK-cell cytotoxicity, enhances antigen presentation by DCs, and modulates macrophage activity [97]. The clinical development of the 4-1BB agonists has been marked by a clear evolution based on past experiences. First-generation agonist mAbs like urelumab initially showed promise but were limited by dose-dependent hepatotoxicity, a significant on-target, off-tumor effect [98]. Subsequent agents, such as utomilumab, aimed to decrease these toxicities by modifying the Fc domain to reduce off-target effects but have demonstrated modest activity in a phase I study (ORR = 3.8%) [99]. New-generation agonists are being developed. Among these, AGEN2373 and ADG206 are IgG1 antibodies with distinct FcγRs affinities. For example, AGEN2373 has demonstrated an ORR of 11% without significant liver toxicity [100]. Similarly, ADG106 and ATOR1017, both IgG4 antibodies, were designed to optimize receptor engagement. ADG106 reported a favorable safety profile (G3/4 TRAEs = 21%), but no objective responses were observed [101]. Other novel compounds such as EU101 (NCT04903873) and YH004 (NCT05564806) are currently under investigation in phase I clinical trials (see Table 2). Recent advancements in bispecific and multispecific antibodies now enable simultaneous targeting of 4-1BB with other ICI or TAAs, offering the potential for more favorable clinical outcomes [102].

### 3.3. Other Co-Stimulatory Receptors

GITR (TNRFS18) and ICOS represent additional co-stimulatory molecules explored as immunotherapeutic targets. GITR is constitutively expressed on Tregs and, at lower levels, on NK cells, T cells, and B cells, with its expression upregulated upon activation [103,104]. Its ligand, GITRL (TNFS18), is primarily found on activated APCs and has also been detected in some tumor cells [105]. GITR activation enhances effector T-cell responses while attenuating the immunosuppressive activity of Tregs, making it an attractive target. Despite promising preclinical data, clinical translation has been challenging. GITR agonists have demonstrated limited efficacy in early phase clinical trials [106]. For instance, TRX518, a GITR agonist, was assessed in a phase I trial, showing an ORR of 3.2% as monotherapy, while the combination with pembrolizumab or nivolumab resulted in ORRs of 4% and 11.2%, respectively. Although the combination showed a modest improvement, response rates remain suboptimal [107].

Meanwhile, ICOS, a CD28 superfamily member, is selectively upregulated on activated T cells and constitutively expressed on Tregs [82]. As a result, ICOS plays a dual role in the TME, promoting effector T-cell activation and antitumor immunity while also supporting Tregs that contribute to immunosuppression [108]. Several ICOS agonists have been evaluated in clinical trials. KY1044 and vopratelimab are IgG1 mAbs designed to deplete Tregs through ADCC, thereby increasing the effector T-cell/Treg ratio within the TME [82]. KY1044 was investigated in a phase I clinical trial in combination with atezolizumab (anti-PD-L1), showing an ORR of 5% [109]. The combination of vopratelimab and the anti-PD-1, nivolumab, was also investigated in the phase I/II ICONIC trial (ORR = 2.3%) [110]. Feladilimab (GSK3359609), an IgG4 mAb, is considered a true agonist due to its lack of Treg-depleting effects and was evaluated in the phase I/II INDUCE-2 trial in combination with the anti-CTLA-4 mAb tremelimumab [111]. The trial reported only a single confirmed partial response (ORR = 4%), ultimately leading to its discontinuation [111]. Overall, these findings highlight the challenges associated with targeting co-stimulatory receptors for cancer immunotherapy and underscore the need for further research to optimize therapeutic strategies.

## 4. Cytokine Modulation

Cytokines are pivotal mediators of the immune system, essential in shaping the TME and modulating innate and adaptive immune responses against cancer. These small and soluble proteins—including interleukins (ILs), interferons (IFNs), TNFs, growth factors, and chemokines—influence key processes such as inflammation, immune cell activation, recruitment, differentiation, and survival [112]. Therapeutic strategies focus on enhancing the immune-activating properties of IL-2, IL-7, IL-12, and IL-15, while also suppressing the pro-inflammatory and tumor-promoting cytokines, such as TNF-α, IL-1β, and IL-6.

### 4.1. Interleukin-2 (IL-2)

Historically, cytokines such as IL-2 and IFN-α were the first immunotherapies approved for cancer treatment. High-dose recombinant IL-2 (aldesleukin) has shown durable responses in subsets of patients with metastatic melanoma and renal cell carcinoma by expanding cytotoxic T cells and NK cells [113,114]. However, these therapies were often limited by significant toxicities, including vascular leak syndrome and a narrow therapeutic window. Furthermore, IL-2 paradoxically induced immunosuppressive Tregs, which counteract its desired immunostimulatory effects [115]. This effect is mediated by the alpha subunit (CD25) of the high-affinity IL-2 receptor (IL-2Rαβγ), which is predominantly expressed on Tregs [116]. By contrast, CD8^+^ T cells, memory T cells, and NK cells predominantly express an intermediate-affinity IL-2 receptor (IL-2Rβγ) that lacks the alpha subunit [117]. To address this issue, novel IL-2 derivatives have been developed to selectively stimulate IL-2Rβγ while minimizing the activation of Tregs. Bempegaldesleukin, also known as NKTR-214, or BEMPEG, is a pegylated IL-2 variant designed to prolong half-life and selectively activate the IL-2Rβγ [118]. In phase I studies, BEMPEG increased PD-1^+^ TILs within the TME, providing a rationale for its combination with anti-PD-1 agents [119]. Despite a manageable safety profile (G3/4 TRAEs = 21.1%) and promising efficacy with an ORR of 59.5% in the phase I/II study PIVOT-02, in combination with nivolumab [119], subsequent phase III trials, including PIVOT IO-001 in melanoma and PIVOT-09 in renal cell carcinoma, failed to demonstrate significant improved efficacy compared to standard therapies [120,121]. These outcomes led to the discontinuation of BEMPEG development. Other non-α-IL2 agonists are currently under investigation. THOR-707 (SAR444245) and MDNA11 are being evaluated in combination with pembrolizumab in their respective phase I clinical trials (NCT04009681 and NCT05086692). Nemvaleukin alfa (ALKS-4230) is an engineered fusion protein comprising IL-2 and the extracellular domain of IL-2Rα, preventing activation of Tregs [122]. The phase I trial, ARTISTRY-1, reported an ORR of 10% for ALKS-4230 as monotherapy and 13% in combination with pembrolizumab in solid tumors [122]. Nemvaleukin alfa is currently being evaluated in the phase III trial ARTISTRY-7 in combination with pembrolizumab for platinum-resistant epithelial ovarian cancer (NCT05092360). Other approaches like AU007, a mAb targeting the CD25-binding epitope of IL-2 [123], and prodrugs such as WTX-124 (NCT05479812) and ODC-IL2 (NCT06770764) are in early stage clinical development (see Table 3).

To reduce systemic toxicity and enhance therapeutic efficacy, engineered IL-2 variants have been combined with antibody-mediated, tumor-targeting strategies. These approaches merge IL-2 with antibodies directed at TAAs (e.g., EpCAM, GD2, CD20, and CEA) or tumor extracellular matrix (ECM) components, enabling selective delivery to the TME and local immune activation [124]. The combination of IL-2 variants (IL-2v) with ICI represents another promising avenue. Immunocytokines, which merge checkpoint inhibition with cytokine-based immune activation, have shown potential in amplifying antitumor responses [125]. For instance, RG6279 (RO7284755) is an immunocytokine that combines a PD-1-blocking antibody and an IL-2v, relying on checkpoint blockade as a central mechanism and amplifying immune activation by targeting the PD-1/PD-L1 axis [126]. A phase I trial is currently ongoing (NCT04303858). Another novel immunocytokine, ANV600, has recently entered the clinical program, taking a novel approach by utilizing a non-blocking PD-1 antibody fused to an IL-2Rβ/γ-selective IL-2v [127]. Unlike RG6279, ANV600 does not inhibit the PD-1/PD-L1 interaction. Instead, the non-blocking PD-1 antibody acts as a targeting moiety, directing IL-2 activity specifically to PD-1-expressing T cells within the TME. This unique design enables ANV600 to synergize with standard anti-PD-1 therapies and is currently being investigated (NCT06470763) [127].

Beyond established IL-2-based therapies, invikafusp alfa (also known as STAR0602) introduces an innovative application of IL-2 in cancer immunotherapy [128]. This first-in-class bsAb targets TCR Vβ receptors, specifically Vβ6 and Vβ10, to selectively activate T cells while concurrently delivering IL-2 via an attached domain on the antibody’s opposing arm [128]. By simultaneously engaging IL-2 receptors and Vβ6/Vβ10 TCRs on the same T cell, invikafusp alfa facilitates the proliferation of both selective CD4^+^ and CD8^+^ T cells [128]. In the phase I/II START-001 trial (NCT05592626), invikafusp alfa showed the most significant efficacy with two confirmed partial responses (ORR = 50%) among heavily pretreated patients, including anti-PD-1/PD-L1 therapy, with high tumor mutational burden (TMB-H) MSS CRC [129]. These promising findings have supported the FDA fast-track designation for patients with advanced TMB-H CRC [130]. The most common TRAEs were transient and low grade [129].

In summary, the therapeutic application of IL-2 has undergone a significant evolution from the systemic administration of high-dose aldesleukin towards highly engineered variants and delivery systems. These newer strategies aim to uncouple the potent antitumor effector functions of IL-2 from its detrimental toxicities and Treg-activating properties. While promising, many of these next-generation approaches are still in early to mid-stage clinical development, and demonstrating a consistently favorable therapeutic index remains a critical ongoing objective.

### 4.2. Interleukin-15 (IL-15)

IL-15 is gaining attention for its ability to expand memory T cells and enhance NK-cell activation [131]. A key rationale for its development is its distinct interaction with shared IL-2/IL-15 receptor components (β and γ chains). IL-15 utilizes its own α-receptor subunit (IL-15Rα) for high-affinity binding and trans-presentation, leading to potent activation of CD8^+^ T cells and NK cells, often without the stimulation of immunosuppressive Tregs commonly seen with wild-type IL-2. For instance, N-803 (ALT-803), an IL-15 superagonist, has demonstrated efficacy in BCG-unresponsive, non-muscle-invasive bladder cancer, reporting a complete response rate of 71% with a median duration of 26.6 months in the QUILT-3.032 phase II/III trial, where it was administered intravesically [132]. This localized success, where systemic exposure is minimal, highlights a potential niche. In contrast, intravenous/subcutaneous administration has been evaluated in solid tumors in a phase I trial, but no clinical activity was observed (ORR = 0%) [133]. Fusion proteins combining IL-15 with its receptor alpha chain (IL-15α) have demonstrated enhanced PK and antitumor efficacy. For example, SOT101 showed encouraging efficacy in the phase I AURELIO-03 trial, with a clinical benefit rate of 63% and an acceptable safety profile [134]. Common toxicities associated with the systemic IL-15 pathway can include transient lymphopenia, cytokine release syndrome-like symptoms, and liver enzyme elevations, generally considered less severe than those associated with high-dose IL-2 but still requiring careful management. Other IL15/IL15α fusion proteins, such as NIZ985 and XmAb306, are being evaluated in phase I trials [124]. Similarly to IL-2, immunocytokines focusing on PD-1/PD-L1, such as SOT201 (NCT06163391) and SAR445877 (NCT05584670), are under investigation in early phase clinical trials.

### 4.3. Other Cytokines

IL-12 boosts antitumor immunity by enhancing T cell and NK cell activity while increasing IFN-γ production, which may potentially convert cold or non-immunogenic tumors into hot tumors. However, systemic toxicities have hindered its clinical application [124]. Strategies such as intratumorally delivered viral or plasmid-based vectors, like the tavokinogene telseplasmid [135] and the engineering of attenuated IL-12 variants, are under investigation. Similarly, while IFNs and TNF-α have demonstrated antitumor potential, systemic toxicities and pleiotropic effects have limited their clinical application. Novel delivery methods, including intratumoral injections and engineered variants, are being explored to harness their benefits with reduced side effects.

## 5. Targeting the Tumor Microenvironment

The TME is a highly dynamic and heterogeneous ecosystem that plays an essential role in tumor progression and immune response modulation. The TME encompasses tumor cells, infiltrating immune cells, cancer-associated fibroblasts (CAFs), endothelial cells, and the ECM. The TME actively influences tumor evolution, immune evasion, therapy resistance, and metastatic dissemination [136]. The dysregulation of the TME contributes to mechanisms such as T-cell exclusion, recruitment of immunosuppressive populations (e.g., Tregs, MDSCs, or TAMs), metabolic reprogramming, and secretion of cytokines that reinforce an immune-excluded phenotype, all of which have been implicated in resistance to current immunotherapies [137]. Given the central role of the TME in immune evasion, multiple therapeutic strategies have emerged to counteract its immunosuppressive effects. While approaches targeting general myeloid-derived cell functions (e.g., CSF1R inhibitors or PI3K inhibitors for TAM reprogramming [138]), CD40 agonists for DC activation [139], or the tumor stroma (e.g., fibroblast activation protein (FAP)-directed therapies) [140] are under investigation, this section will focus on selected additional targets of increasing interest: C-C motif chemokine receptor 8 (CCR8), as a chemokine receptor selectively enriched in intratumoral Tregs [141]; CD47, a key “don’t eat me” signal that enables tumor cells to evade macrophage-mediated phagocytosis [142]; and TGF-β, a potent immunosuppressive cytokine that reinforces stromal fibrosis and T-cell exclusion [143]. These targets are currently being explored in early phase clinical trials and represent promising approaches for reprogramming the TME and improving immunotherapy outcomes in solid tumors.

### 5.1. Depleting Tregs with Anti-CCR8 Therapies

CCR8 is a G-protein-coupled receptor predominantly expressed on Tregs, particularly those infiltrating the TME [144]. Its primary ligand is CCL1, although both CCL8 and CCL18 have been proposed as potential ligands [145]. Intratumoral CCR8^+^ Tregs exhibit enhanced immunosuppressive activity compared to peripheral Tregs, making CCR8 an attractive target for selectively depleting tumor-infiltrating Tregs while preserving systemic immune homeostasis [146]. Furthermore, elevated CCR8 expression has been reported in multiple malignancies, including NSCLC, CRC, melanoma, and breast cancer, where it correlates with poor prognosis [147]. Preclinical studies have explored the role of CCR8 and have demonstrated that, although CCR8 serves as a marker of highly suppressive intratumoral Tregs, CCR8 knock-out mouse models do not exhibit impaired tumor Treg infiltration or a diminished immunosuppressive function suggesting that CCR8 is not essential for Treg trafficking but rather identifies a subset of activated and tumor-resident Tregs [146]. Consequently, blocking CCR8 signaling alone may not be sufficient to enhance antitumor immunity, positioning ADCC-enhancing, anti-CCR8 mAbs as a more effective strategy to deplete these potent immunosuppressive cells [148]. Prior evidence suggests that anti-CCR8 mAbs may selectively deplete intratumoral Tregs, enhance CD8^+^ T-cell activation, and significantly inhibit tumor growth [149]. Unlike other chemokines, such as CCR4, which is broadly expressed on Tregs in both tumoral and peripheral tissues [150], CCR8 expression appears to be largely restricted to tumor-infiltrating Tregs, potentially making it a safer immunotherapeutic target. Furthermore, combining CCR8-targeting antibodies with ICI, such as anti-PD-1, has exhibited synergistic effects, suggesting that a CCR8 blockade may help overcome resistance to current immunotherapies [148]. Early clinical data are emerging. CCR8-targeting antibody LM-108 showed an ORR of 5.2% as a monotherapy in a phase I trial (NCT05255484), with an excellent safety profile. Notably, in combination with pembrolizumab for gastric cancer, an ORR of 36.1% was reported at the 2024 ASCO annual meeting, with G3/4 TRAEs in 37.5% of patients [151]. Interestingly, patients with high CCR8 expression showed a higher ORR (87.5%), providing evidence of on-target immune modulation consistent with the preclinical rationale [151]. Multiple phase I clinical trials are currently ongoing to evaluate anti-CCR8 mAbs, primarily in combination with anti-PD-1/PD-L1 therapies (see Table 4).

### 5.2. Blocking the “Don’t Eat Me” Signal: CD47

CD47 has emerged as a promising therapeutic target due to its role as a myeloid immune checkpoint [152]. CD47 is a transmembrane protein that functions as a “don’t eat me” signal, preventing phagocytosis of healthy cells such as erythrocytes and platelets by interacting with signal regulatory protein alpha (SIRPα), which is expressed on macrophages, DCs, and neutrophils [153,154]. However, CD47 is frequently overexpressed in multiple malignancies, enabling tumor cells to promote immune evasion [155]. Notably, high CD47 mRNA expression has been associated with poor prognosis [156]. Blocking the CD47–SIRPα interaction using mAbs enhances macrophage-mediated phagocytosis of tumor cells, thereby suppressing tumor growth. Several therapeutic approaches are being explored. Magrolimab (Hu5F9-G4), an IgG4 anti-CD47 mAb, has been one of the most advanced agents in clinical development. Initially demonstrating significant efficacy in hematologic malignancies [157], its application in solid tumors has been explored in combination with ICI and chemotherapy. A phase Ib trial that assessed magrolimab in combination with avelumab reported one partial response (ORR = 4.7%) in platinum-resistant or refractory ovarian cancer [158]. However, its clinical progress has been hampered by hematologic toxicities, particularly anemia, due to CD47 expression on red blood cells [159]. While dose-modification strategies such as dose priming have been attempted, anti-CD47 mAbs safety profile remains a concern [160]. For instance, the phase III ENHANCE 3, which evaluated magrolimab in combination with venetoclax/azacitidine, was stopped in hematologic malignancies due to lack of efficacy and safety concerns reporting a higher rate of grade 5 adverse events (15.2%), compared to 9.5% in the control arm, mainly driven by infections and respiratory failure [161]. Clinical trials testing other anti-CD47 mAbs such as IMC002 (NCT05276310), STI-6643 (NCT04900519), and HMPL-A83 (NCT05429008) are currently ongoing (see Table 4). To reduce toxicity and improve therapeutic efficacy, anti-SIRPα mAbs, such as DS-1103a, are currently being explored. Since SIRPα is not expressed on red blood cells, it is suggested that hematologic toxicity may be reduced. Rational combinations with the addition of other targeted mAbs or bsAbs have been postulated to synergize with an anti-CD47/SIRPα blockade by enhancing phagocytosis via ADCC or ADCP [160]. For instance, AK117 (ligufalimab), a humanized IgG4 mAb targeting CD47, demonstrated encouraging antitumor activity and a favorable safety profile. AK117 is structurally optimized to avoid red blood cells’ hemagglutination and minimize hematologic toxicity [162]. In a phase II study of metastatic triple-negative breast cancer, the combination of AK117 with ivonescimab (a PD-1 × VEGF bsAb) achieved an ORR of 72.4% and a mPFS of 9.3 months [163]. Similarly, in HER2-negative advanced gastric or gastroesophageal junction adenocarcinoma, the combination of AK117 with cadonilimab (a PD-1 × CTLA-4 bsAb) and chemotherapy yielded a 75% ORR [164]. Notably, in PD-L1-positive recurrent/metastatic HNSCC, adding AK117 to ivonescimab improved the ORR from 40% to 65% and prolonged mPFS to 7.1 months, supporting the pivotal phase III trial (AK117-302) [165]. These clinical data suggest that an optimized CD47 blockade, especially when combined with ICI, may enhance antigen presentation by promoting macrophage-mediated phagocytosis and subsequent presentation of tumor antigens to T cells [160]. Furthermore, recent advances in antibody engineering have facilitated the development of fusion proteins and bsAbs, offering a promising avenue for improving CD47-targeted therapies and overcoming tumor immune evasion.

### 5.3. Inhibiting TGF-β Signaling

TGF-β plays a key role in immune suppression, stromal remodeling, and resistance to immunotherapy [166]. Initially identified for its role in cell growth regulation, it is now recognized as a pleiotropic cytokine involved in tissue homeostasis, immune regulation, and tumor progression [167]. Tumors exploit TGF-β signaling to evade immune responses, promote Treg expansion, and activate CAFs, contributing to extracellular matrix remodeling and fibrosis [166,168].

Despite promising preclinical data, clinical translation has been difficult due to toxicity and the need to preserve TGF-β’s physiological functions. Many inhibitors lack isoform specificity, potentially leading to adverse effects, particularly cardiovascular and fibrosis [169]. TGF-β receptor type I (TGFβRI) small-molecule inhibitors have been explored with Galunisertib (LY2157299), Vactosertib (EW-7197), LY3200882, GFH018, or YL-13017, showing different profiles in clinical development [170]. Galunisertib, a first-in-class inhibitor, demonstrated a manageable safety profile but limited efficacy in pancreatic cancer (ORR = 4.3%) [171] or hepatocellular cancer (ORR = 0%) [172], leading to discontinued development. Vactosertib, a more potent and bioavailable inhibitor, is being explored in several settings [173], particularly in combination with ICI, chemotherapy, and targeted agents [170]. LY3200882, a next-generation inhibitor, demonstrated a remarkable ORR of 50% in pancreatic cancer patients when combined with gemcitabine and paclitaxel, along with durable responses in glioblastoma, supporting its continued clinical investigation [174]. MAbs targeting TGF-β have also been investigated. Fresolimumab (GC-1008), a pan-TGF-β mAb that blocks all three isoforms, showed modest antitumor responses in various cancers [175]. Notably, Fresolimumab has been associated with the development of treatment-emergent skin lesions including keratoacanthomas and squamous-cell carcinomas, which remains an important toxicity concern [176]. Other mAbs targeting the TGF-β pathway, such as NIS793, SAR439459, and SRK-181, have been developed (see Table 4). For instance, SRK-181, a selective inhibitor of latent TGF-β1, was evaluated in combination with pembrolizumab in the phase I DRAGON trial (NCT04291079). The combination showed an ORR of 33.3% in HNSCC and 20% in both melanoma and clear renal cell carcinoma, anti-PD-1-resistant tumors, thus showing promise in overcoming resistance to PD-1 blockade [177]. This approach is based on the critical role of TGF-β1 in tumor progression, as it is the predominant isoform involved in immunosuppression and resistance to ICI [178]. Recent strategies have shifted toward developing fusion proteins and bispecific agents that offer enhanced selectivity and dual-targeting approaches. These novel strategies aim to mitigate off-target effects while improving antitumor efficacy by simultaneously modulating TGF-β and other key immunosuppressive pathways. BsAbs, such as bintrafusp alfa (M7824), which targets both TGF-β and PD-L1, have shown preclinical and early clinical data, leveraging a dual blockade to restore immune surveillance in resistant tumors. In clinical studies, bintrafusp alfa demonstrated an ORR of 21.3% in a phase I trial for previously treated NSCLC [179] and 21.9% in post-platinum-therapy cervical cancer [180], but only an ORR of 10.7% in a phase II trial for biliary tract cancer [181]. However, despite promising early results, bintrafusp alfa failed in a phase III trial (NCT03631706) to demonstrate superiority over anti-PD-1 monotherapy in high PD-L1-expressing advanced NSCLC patients [182]. Another bifunctional fusion antibody is BCA101, a first-in-class agent that simultaneously targets the epidermal growth factor receptor (EGFR) and TGF-β signaling [183]. A phase 1/1b clinical trial evaluated BCA101 as a monotherapy and in combination with pembrolizumab, demonstrating an ORR of 44% in recurrent or metastatic HNSCC [184]. Similarly, fusion proteins engineered to sequester TGF-β ligands (ligand traps) or selectively inhibit signaling in the TME are being investigated to overcome immune suppression while preserving physiological TGF-β functions [185]. Despite the promise of these next-generation agents, optimizing patient selection and identifying predictive biomarkers remain crucial for their successful clinical implementation.

### 5.4. Targeting Metabolic Pathways

In addition to the previously mentioned strategies, targeting metabolic pathways within the TME has been explored. Although preclinical studies have underscored the importance of metabolic reprogramming in tumor progression and immune evasion, the translational progress of metabolic interventions has been impeded by challenges such as target specificity, metabolic redundancy, and the complexity of compensatory pathways [186]. An example is the development of indoleamine 2,3-dioxygenase (IDO) inhibitors, which were initially raised for their potential to counteract tumor-induced immunosuppression by disrupting tryptophan metabolism. Despite robust preclinical data, IDO inhibitors have encountered significant difficulties in clinical development. For instance, in the phase 1 trial (NCT01195311), which evaluated epacadostat as a single agent, no objective responses were observed [187]. However, its combination with the anti-PD-1 pembrolizumab demonstrated clinical activity (ORR = 55%) in the phase I/II trial ECHO-202/KEYNOTE-037 [188]. Nevertheless, epacadostat, an IDO1 inhibitor, failed to meet its primary endpoints in a pivotal phase III trial (ECHO-301/KEYNOTE-252) on advanced melanoma patients, thereby dampening enthusiasm for this class of metabolic therapeutics [189]. Similarly, the adenosine pathway plays a role in immunosuppression within the TME. The adenosine pathway suppresses T-cell activation and promotes exhaustion by accumulating adenosine in the TME through CD39/CD73, which activates A2A receptors on T cells. For example, oleclumab, a first-in-human anti-CD73 mAb, in combination with anti-PD-L1 durvalumab, showed an ORR of 2.4%, 4.8%, and 9.5% in the pancreatic and NSCLC expansion cohorts of the phase I trial (NCT02503774) [190]. Moreover, progress in metabolomic profiling may provide new insights into the roles of metabolic processes in tumor immunity.

## 6. Exploring Novel Targets with Bispecific Antibodies

The advent of bsAbs marks a new era in cancer immunotherapy. They offer a novel approach for targeting malignancies through their ability to bind two distinct antigens or epitopes simultaneously. Unlike conventional mAbs, bsAbs may enhance therapeutic efficacy by engaging immune effector cells, modulating oncogenic signaling pathways, or overcoming resistance mechanisms within the TME.

The versatility of bsAbs lies in their structural diversity and functional adaptability. Structurally, bsAbs can be classified based on the presence or absence of an Fc region and the configuration of their antigen-binding domains [191]. IgG-like bsAbs retain the Fc region, which prolongs half-life and enables Fc-mediated functions such as ADCC [192]. However, their larger size may limit tumor penetration and increase off-tumor toxicity [193]. To address these issues, strategies such as using IgG subclasses with reduced FcγR binding (e.g., IgG2 or IgG4 instead of IgG1 or IgG3) or introducing specific Fc mutations to silence Fc activity have been explored [194,195]. Non-IgG-like bsAbs, such as BiTEs and DART molecules, lack an Fc domain, offering enhanced tumor penetration in the TME. However, they exhibit shorter half-lives, which can be mitigated by albumin fusions, pegylation, or increasing the frequency of infusion. Depending on the therapeutic goal, either format may be preferable [196,197]. For example, non-IgG-like bsAbs are particularly advantageous in applications like T-cell engagers or checkpoint modulation, where systemic immune activation must be minimized. Conversely, enhancing Fc interactions, such as increasing FcγRIIIa binding, can promote antitumor activity via ADCC, especially for bsAbs targeting oncogenic signaling pathways. Beyond the Fc region, other structural features, such as valency and specificity of epitope binding, can also be optimized [191]. This structural and functional flexibility has raised bsAbs to the forefront of next-generation cancer therapies, enabling tailored approaches to overcome tumor heterogeneity, immune evasion, and resistance mechanisms. Functionally, bsAbs can be classified into three major categories: immune cell engagers (ICEs), checkpoint modulators, and signal blockers. We will focus primarily on the first two categories—ICEs and checkpoint modulators—due to their impact on the immuno-oncology field and early drug development. ICEs are designed to recruit and activate immune effector cells, such as T cells or NK cells, by bringing them into close proximity with tumor cells through TAA targeting. This mechanism potentially induces cytotoxicity independently of MHC-restricted antigen presentation [198]. While T-cell engagers (TCE) have demonstrated remarkable efficacy in hematological malignancies, as seen with blinatumomab (CD19 × CD3) in B-cell leukemia [199], their application in solid tumors has been challenging. Key barriers include limited T-cell infiltration, an immunosuppressive TME, and potential on-target, off-tumor toxicity due to antigen expression in normal tissues.

Cell surface antigens (e.g., HER2, EGFR, EPCAM, and CEACAM5, among others) are being tested in multiple clinical trials, but some challenges have emerged. For instance, solitomab, an EPCAM × CD3 bsAbs, showed > 95% of G3 TRAEs due to off-tumor toxicity due to EPCAM expression in non-tumor tissues. Consequently, its clinical development was discontinued [200]. Targeting specific-tissue antigens, like prostate-specific membrane antigen (PSMA), may reduce off-tumor toxicity as demonstrated by pasotuxizumab (PSMA × CD3) which has shown a favorable safety profile and early efficacy signs by prostate-specific antigen (PSA) reductions and durable responses in metastatic castration-resistant prostate cancer (mCRPC) [201]. Interestingly, subcutaneous infusion was associated with the development of anti-drug antibodies, potentially limiting its efficacy in this cohort [201]. Additional PSMA-targeted TCEs are under clinical evaluation, both as monotherapies and in combination with ICI. This is the case of REGN4336 (NCT05125016). Other therapeutic strategies have been developed to enhance tumor selectivity and decrease toxicity. For instance, conditionally active biologics (CAB), such as BA3182 (CAB–EPCAM × CAB–CD3), are designed to be activated within the acidic TME, thereby reducing systemic toxicity. BA3182 is currently under evaluation in a phase I trial (NCT05808634). A novel therapeutic approach is the use of masked antibodies developed to prevent binding to their target in normal tissues [202]. The masking element, often a peptide or a protein domain, is strategically placed to cover the antibody’s binding site and designed to be removed specifically within the TME by tumor-associated proteases or specific biochemical conditions [202]. For instance, VIR-5818 (previously SAR4463309), a dual-masked HER2-targeting TCE, is being investigated in a phase I clinical trial (NCT05356741) for patients with HER2-expressing solid tumors. Preliminary data suggest promising antitumor activity, particularly in HER2-positive CRC, with a partial response rate of 33% [203]. Notably, no grade ≥ 3 CRS cases have been reported [203]. Similarly, VIR-5500, a PSMA-targeting dual-masked TCE, is in phase I evaluation for mCRPC (NCT05997615) [203]. Early findings indicate PSA reductions in all patients treated in the study, with no DLTs observed [203]. Targeting tumor-selective antigens, which are overexpressed on malignant cells but present at minimal levels in normal tissues, could also be a promising approach. These antigens, such as DLL3, claudin 18.2, claudin 6, ROR1, or STEAP1, also offer a promising approach. Indeed, tarlatamab (DLL3 × CD3 TCE) has emerged as a therapeutic option in SCLC. The phase II DeLLphi-301 trial reported an ORR = 32–40% with tarlatamab in previously treated SCLC patients, leading to FDA approval in 2024 [204]. Ongoing phase III trials, including DeLLphi-304 (NCT05740566), DeLLphi-305 (NCT06211036), and DeLLphi-306 (NCT06117774), are further evaluating its efficacy in relapsed, first-line extensive-stage, and limited-stage SCLC, respectively, to define its role in earlier treatment settings.

Beyond surface antigens, recent advances have focused on targeting intracellular tumor antigens via MHC presentation. Immune mobilizing monoclonal TCRs against cancer (ImmTACs) are designed to exploit the high specificity of TCRs for recognizing processed peptides derived from intracellular proteins presented by MHC molecules [205]. Unlike classical mAbs that recognize surface antigens, ImmTACs recognize intracellularly processed peptides displayed on HLA-A*02:01. Notably, tebentafusp, which targets gp100 in uveal melanoma, has demonstrated an OS benefit in a phase III trial, leading to regulatory approval [206]. Beyond ImmTACS, novel TCR-based approaches are emerging. Recently, two phase I trials have been initiated to evaluate a next-generation T-cell Engaging Receptor (TCER^®^), which combines a high-affinity TCR domain against an HLA-A*02:01-presented MAGEA4/8 peptide (NCT05359445) and PRAME (NCT05958121). Beyond conventional αβ T cells, γδ T cells, which recognize antigens in an MHC-independent manner, represent alternative T-cell populations and could overcome some resistance mechanisms such as low expression of MHC [207]. This new approach is being tested in early clinical trials such as LAVA-1207, which binds with high affinity to the Vδ2 chain of Vγ9Vδ2-T cells and to PSMA, in monotherapy and in combination with IL-2 or pembrolizumab (NCT05369000). In addition to T-cell-based therapies, bispecific killer cell engagers (BiKEs) target CD16 (FcγRIIIa), a key activating receptor on NK cells, to enhance ADCC [208]. A leading candidate in solid tumors is AFM24 (EGFR × CD16A), which engages NK cells to attack EGFR-expressing tumors, such as NSCLC and CRC. Data from ASCO 2024 showed that AFM24, particularly in combination with atezolizumab, achieved an ORR of 26.7% and a mPFS of 5.9 months in EGFR wild-type NSCLC patients who had progressed after chemotherapy and immunotherapy [209].

In contrast to T-cell engagers, checkpoint-modulating bAbs aim to enhance immune responses by blocking multiple immune inhibitory checkpoints simultaneously or combining checkpoint blockade with immune co-stimulation [210]. Unlike conventional ICI, bispecific checkpoint inhibitors can target two inhibitory pathways at once, potentially preventing immune escape and T-cell exhaustion. While combination therapy with PD-1/L1 and CTLA-4 inhibitors has proven effective in some cancers, bsAbs may offer improved PK, reduced systemic toxicity, and greater tumor-specific immune modulation [191]. Notably, cadonilimab, first-in-class PD-1 × CTLA-4 bsAb, has demonstrated strong clinical efficacy in phase III clinical trials, leading to regulatory approval in China for relapsed/metastatic cervical cancer and advanced gastric and gastroesophageal cancer [211,212]. Further global investigations will be essential to confirm its broader applicability. Other promising anti-PD-1 × CTLA-4 bsAbs, including lorigerlimab and volrustomig, are under evaluation in monotherapy and in combination with antiangiogenics (NCT04522323) or ADCs (NCT05293496). Beyond PD-1/CTLA-4, resistance to PD-1 inhibitors is often mediated by compensatory upregulation of LAG-3, TIM-3, or TIGIT. Ongoing clinical trials are evaluating bsAbs that simultaneously inhibit these pathways (see Table 5).

Dual co-stimulatory bsAbs have also been used to enhance antitumor responses while limiting the toxicity observed with agonist antibodies, which need crosslinking via FcγR to become active [213]. In this regard, FS-120 (4-1BB × OX40) has been designed to be FcγR-null and, instead, to utilize bispecific crosslinking to induce strong receptor clustering and activation without engaging FcγR, potentially limiting systemic toxicity [213]. Building upon this concept, co-stimulatory bsAbs have been engineered to further enhance tumor-specific immune activation. This approach aims to selectively recruit T cells that express co-stimulatory receptors within the TME rather than indiscriminately activating all T cells [207]. Similar to ICEs, these bsAbs are designed with one arm recognizing a TAA such as EGFR, HER2, or PSMA, among others, while the other arm targets a co-stimulatory receptor like OX40, 4-1BB, CD40, or CD28 (see Table 5) with the ultimate goal of minimizing off-target toxicity. For instance, FS222 is a bsAbs targeting PD-L1 while concurrently engaging 4-1BB, thereby facilitating localized T-cell co-stimulation within the TME, particularly in regions of high PD-L1 expression [102]. In the phase I trial (NCT04740424), FS222 (4-1BB × PD-L1) showed an ORR of 17% across all tumor types. Notably, among patients with previously ICI-treated melanoma, the ORR reached of 47.4% in previously PD-1 mAb-treated melanoma patients [102]. G3/4 TRAEs occurred in 36% of patients, although none led to treatment discontinuation [102].

By combining the CD47 blockade with a tumor-specific antigen (e.g., CD47 × DLL3, CD47 × HER2, or CD47 × CLDN 18.2), it may be possible to selectively restore macrophage-driven tumor clearance while minimizing systemic toxicity associated with global CD47 inhibition. This approach not only enhances innate immune activation but may also create a more favorable immune TME, ultimately aiming to improve the efficacy of T-cell-based immunotherapies. Apart from TAA, bsAbs can target both PD-1 and CD47. They are being explored to overcome adaptive and innate immune resistance mechanisms simultaneously [214]. PD-1 × CD47 bsAbs offer a coordinated approach to enhance both T-cell cytotoxicity and macrophage-driven tumor clearance, potentially leading to synergistic antitumor activity compared to monotherapies [215]. This dual inhibition strategy may be particularly advantageous in tumors with both T-cell exhaustion and myeloid-driven immune suppression, offering a novel avenue to enhance responses in immune-resistant cancers.

## 7. Current Challenges and Future Directions

Advancements in cancer immunobiology and the evolution of drug development platforms have led to a rapid expansion in the discovery of immunotherapy targets, the development of novel agents, and the initiation of numerous clinical trials evaluating their efficacy [22]. Recognizing the most promising targets and therapeutic agents to maximize antitumor activity while minimizing off-target adverse events will be essential for accelerating the translation of discoveries into long-term clinical benefits for patients. While ICI have revolutionized cancer treatment, resistance mechanisms remain a challenge. To overcome these limitations, novel therapies targeting LAG-3, TIM-3, TIGIT, and co-stimulatory receptors like OX40 and 4-1BB, as well as strategies to modulate the TME, have been examined.

Nevertheless, successfully advancing agents against these novel targets requires navigating substantial development obstacles. For instance, the experience with co-stimulatory agonists illustrate these difficulties, highlighting how initial setbacks can drive crucial mechanistic insights and refine therapeutic strategies. The clinical translation of co-stimulatory agonists, despite strong preclinical foundations, was significantly challenged by modest monotherapy efficacy [89,90,91], and dose-limiting toxicities [98]. Subsequent research highlighted the critical role of Fc domain modifications in both therapeutic activity and safety. The choice of IgG isotype and specific mutations to modulate FcγR interactions are now recognized as crucial for optimizing receptor clustering, agonistic signaling, and mitigating off-target effects or unintended cell depletion. This understanding, coupled with optimized dosing, scheduling, and rational combination partners, has driven the development of new co-stimulatory agents.

It is now widely acknowledged that components of the TME can significantly impact drug penetration, distribution, metabolism, and overall therapeutic response [216]. For instance, immune suppressive cells within the TME may negatively impact responses to ICI, while the surrounding stromal elements can hinder the chemotherapy delivery, contributing to drug resistance [217,218]. For this reason, it is increasingly accepted that TME can function as a biomarker for predicting the response to treatment [219]. Intratumoral heterogeneity (ITH) and immune editing represent significant challenges in developing novel immunotherapies. ITH describes the coexistence of distinct subclonal populations within a tumor, which can respond differently to treatment, leading to dissociated responses [220]. ITH includes variations in immune composition across different tumor locations, disease stages, and even distinct regions within the same tumor. Furthermore, sites of metastatic spread can critically influence immune responses. A well-documented example is MSS CRC with liver metastases, where the hepatic microenvironment fosters immune tolerance, diminishing the efficacy of ICI [221,222]. These complexities underscore the need for novel immunotherapeutic strategies capable of addressing ITH-driven resistance and immune evasion. The TME is a highly dynamic and complex ecosystem composed of immune cells, stromal components, blood vessels, and extracellular matrix elements that interact with tumor cells to influence disease progression and response to therapy [223]. Tumor–host interactions extend beyond the cellular level, involving intricate signaling networks, metabolic crosstalk, and immune modulation that can either support or suppress tumor growth [224,225]. Understanding these multifaceted interactions will be essential for identifying novel therapeutic targets within the TME. Efforts to develop new immunotherapeutic strategies are increasingly focused on modulating key components of the TME, aiming to overcome immune evasion, enhance antitumor immunity, and improve treatment efficacy across different cancer types.

The importance of combination immunotherapy in early phase clinical trials lies in its potential to overcome primary and prevent acquired resistance mechanisms seen in monotherapies, thus broadening the number of patients who benefit from treatment. However, these trials are inherently complex due to the vast number of possible drug combinations, varying mechanisms of action, and the need for optimized dosing strategies to balance efficacy and safety. Challenges include patient selection, biomarker integration, and the development of novel trial designs that allow for adaptive dosing and early efficacy assessment. Strategic, biomarker-driven approaches and innovative trial methodologies will be crucial for successfully translating combination immunotherapies into clinical practice [226]. As previously discussed, advancing immunotherapy requires overcoming key challenges, including ITH, the complexity of the TME, tumor–host interactions, immune editing, dissociated responses, and the need for better patient selection through novel predictive biomarkers and resistance mechanisms. Cutting-edge technologies will be essential to address these issues. Single-cell approaches like scRNA-seq provide unprecedented insights into immune heterogeneity, reshaping drug discovery [227]. Spatial transcriptomics, proteomics, and metabolomics offer a deeper understanding of the TME interactions while preserving spatial context [227]. Furthermore, CRISPR-based screening may also identify therapeutic targets and resistance mechanisms [228], while the integration of Artificial Intelligence (AI) offers powerful tools to optimize drug development [229,230]. As novel immunotherapy targets are explored, new patterns of immune-related adverse events have emerged, posing significant challenges for patient management. Several mitigation strategies have been proposed, such as patient education and monitoring, standardized management guidelines, early detection, optimized immunosuppressive therapies, personalized treatment plans, integration of advanced technologies, and predictive biomarkers [231,232,233]. Some of the plausible biomarkers studied for this purpose include high granularity peripheral blood phenotyping, circulating cytokines and chemokines, autoantibodies, and the composition of gut microbiota [234]. Also, the integration of high-throughput bioinformatic “omics” technologies into the understanding of immunotherapy-related adverse effects will become increasingly common and essential in clinical research [234].

In conclusion, the development of novel immunotherapy agents and combination strategies to overcome ICI resistance is progressing rapidly, with numerous clinical trials currently underway. However, identifying the optimal immunotherapy regimen for each tumor type and patient remains a challenge. Personalized treatment strategies, informed by biomarkers for patient stratification, offer the potential to overcome resistance and reduce immune-related adverse events, ultimately improving outcomes. While early signs of activity provide hope for improving responses in non-immunogenic cancers, a deeper understanding of toxicity profiles and patient selection strategies will be crucial to optimize patient outcomes.

## Figures and Tables

**Table 1:** Selected active trials involving inhibitory checkpoint inhibitors. This is a non-exhaustive, manually curated list intended to highlight ongoing studies of clinical relevance.

Drug Type	Mechanism	Trial ID	Drugs Combination	Phase	Tumors	Status	Results
LAG-3							
Relatlimab (BMS-986016)	Antagonist mAb	NCT01968109	Single therapy, anti-PD-1 (nivolumab)	1/2a	Advanced malignancies	Active, not recruiting	Cohort C: ORR = 16%; Cohort D: ORR = 9.2–12.1%
		NCT03044613	Anti-PD-1 (nivolumab) and CRT	1	Neoadjuvant esophageal/GEJ carcinoma	Active, not recruiting	Arm B: pCR = 21.4%
		NCT04658147	Anti-PD-1 (nivolumab)	1	Resectable HCC	Recruiting	n/a
		NCT05337137	Anti-PD-1 (nivolumab), bevacizumab	1	Advanced HCC	Active, not recruiting	n/a
		NCT06683755	Anti-PD-1 (nivolumab), anti-CTLA-4 (ipilimumab)	1/2	Advanced Melanoma	Not yet recruiting	n/a
INCAGN02385	Antagonist mAb	NCT04370704	Anti-PD-1 (retinfalimab), anti-TIM-3 (INCAGN02390)	1/2	Advanced malignancies	Active, not recruiting	n/a
LBL-007	Antagonist mAb	NCT03744468	Anti-PD-1 (tislelizumab), anti-TIM-3 (BGB-A425)	1/2	Advanced malignancies	Active, not recruiting	n/a
		NCT05102006	Anti-PD-1 (toripalimab)	1/2	Advanced malignancies	Recruiting	ORR = 13.3%; DCR = 48.0%
TQB2223	Antagonist mAb	NCT05894421	Anti-PD-1 (penpulimab)	1	Advances malignancies	Recruiting	n/a
		NCT06320080	Anti-PD-1 (penpulimab)	1	Advanced HCC	Recruiting	n/a
IBI110	Antagonist mAb	NCT06494943	Anti-PD-1 (sintilimab)	1	LA HNSCC	Active, not recruiting	n/a
Eftilagimod alpha (IMP321)	Soluble fusion protein	NCT03252938	Single therapy, CT, or avelumab	1	Advanced malignancies	Recruiting	n/a
TIM-3							
BC4302	Antagonist mAb	NCT06608940	Anti-PD-L1 (durvalumab), anti-CTLA-4 (tremelimumab)	1/2	Advanced HCC	Not yet recruiting	n/a
Cobolimab (TSR-022)	Antagonist mAb	NCT02817633	Single therapy, anti-PD-1 (nivolumab/dostarlimab)	1	Advanced malignancies	Recruiting	n/a
Sabatolimab (MBG453)	Antagonist mAb	NCT03961971	Anti-PD-1 (spartalizumab)	1	Recurrent GBM	Active, not recruiting	n/a
INCAGN02390	Antagonist mAb	NCT04370704	Anti-PD-1 (retifanlimab), anti-LAG-3 (INCAGN02385)	1/2	Advanced melanoma	Active, not recruiting	n/a
TIGIT							
COM902	Antagonist mAb	NCT04354246	Single therapy, anti-PVRIG (COM701), anti-PD-1 (pembrolizumab)	1	Advanced malignancies	Recruiting	MSS-CRC cohort: ORR 5%; DCR 40%.
Tiragolumab	Antagonist mAb	NCT05394337	Anti-PD-L1 (atezolizumab)	1/2	Neoadjuvant UC	Recruiting	n/a
Domvanalimab	Antagonist mAb	NCT04656535	Anti-PD-1 (zimberelimab)	0/1	GBM	Recruiting	n/a
PM1021	Antagonist mAb	NCT05537051	Single therapy, PD-1 × TGF-β (PM8001)	1	Advanced malignancies	Not yet recruiting	n/a
Tamgiblimab (IBI939)	Antagonist mAb	NCT04353830	Single therapy, anti-PD-1 (sintilimab)	1	Advanced malignancies	Not yet recruiting	n/a
Belrestotug (EOS-448)	Antagonist mAb	NCT05060432	Single therapy, anti-PD-1 (pembrolizumab or dostarlimab), A2AR inhibitor (inupnant), chemotherapy	1/2	Advanced malignancies	Active, not recruiting	n/a
AB308	Antagonist mAb	NCT04772989	Anti-PD-1 (zimberelimab)	1	Advanced malignancies	Active, not recruiting	n/a
AK127	Antagonist mAb	NCT05868876	AK104 (PD-1 × CTLA-4)	1	Advanced malignancies	Recruiting	n/a
HLX53	Fc fusion protein	NCT05394168	Single therapy, anti-PD-1 (nivolumab)	1	Advanced malignancies	Active, not recruiting	n/a
B7-H5							
HMBD-002	Antagonist mAb	NCT05082610	Anti-PD-1 (pembrolizumab)	1	Advances malignancies	Recruiting	n/a
SNS-101	Antagonist mAb	NCT05864144	Anti-PD-1 (cemiplimab)	1/2	Advances malignancies	Recruiting	ORR = 3%; DCR = 35%
PMC-309	Antagonist mAb	NCT05957081	Anti-PD-1 (pembrolizumab)	1	Advances malignancies	Not yet recruiting	n/a

n/a = not available data (ongoing or recently completed trial); mAb: monoclonal antibody; CT: chemotherapy; CRT: chemoradiotherapy; GEJ: gastroesophageal junction; HCC: hepatocellular carcinoma; LA: locally advanced; HNSCC: head and neck squamous cell cancer; MSS-CRC: microsatellite-stable colorectal cancer; UC: urothelial carcinoma; GBM: glioblastoma multiforme; ORR: overall response rate; DCR: disease control rate; pCR: pathological complete response.

**Table 2:** Selected active trials involving co-stimulatory receptors. This is a non-exhaustive, manually curated list intended to highlight ongoing studies of clinical relevance.

Drug Type	Mechanism	Trial ID	Drugs Combination	Phase	Tumors	Status	Results
OX40							
INBRX-106	Agonistic mAb	NCT04198766	Anti-PD-1 (pembrolizumab)	1/2a	Advanced malignancies	Recruiting	n/a
BGB-A445	Agonistic mAb	NCT04215978	Anti-PD-1 (tislelizumab)	1/2	Advanced malignancies	Active, not recruiting	ORR = 23%; DCR 66.7%
	Agonistic mAb	NCT05661955	Anti-PD-1 (tislelizumab)	1	Advanced UC, RCC, melanoma	Recruiting	n/a
ES102	Agonistic mAb	NCT04730843	Anti-PD-1 (toripalimab)	1	Advanced malignancies	Recruiting	n/a
HFB301001	Agonistic mAb	NCT06623136	Single therapy	1	Advanced malignancies	Active, not recruiting	DCR > 60%
HLX51	Agonistic mAb	NCT05788107	Single therapy	1	Advanced malignancies	Not yet recruiting	n/a
GEN1055	Agonistic mAb	NCT06391775	Anti-PD-1 (pembrolizumab)	1	Advanced malignancies	Recruiting	n/a
4-1BB	(CD137)						
YH004	Agonistic mAb	NCT05564806	Single therapy	1	Advanced malignancies	Recruiting	n/a
EU101	Agonistic mAb	NCT04903873	Single therapy	1/2	Advanced malignancies	Recruiting	n/a
ADG206	Agonistic mAb	NCT05614258	Single therapy	1	Advanced malignancies	Recruiting	n/a
LVGN6051	Agonistic mAb	NCT05301764	TKI (anlotinib)	1/2	Soft-tissue sarcomas	Recruiting	ORR = 6.9%; DCR 86.2%; G3–4 TRAEs: 61.54%
GITR							
REGN6569	Agonistic mAb	NCT04465487	Anti-PD-1 (cemiplimab)	1	Advanced malignancies	Active, not recruiting	ORR = 6.9%

n/a = not available data (trial ongoing or recently completed trial); mAb: monoclonal antibody; TKI: tyrosine-kinase inhibitor; UC: urothelial carcinoma; RCC: renal cell carcinoma; ORR: overall response rate; DCR: disease control rate; G3/4 TRAEs: grade 3–4 treatment-related adverse events.

**Table 3:** Selected active trials involving cytokines. This is a non-exhaustive, manually curated list intended to highlight ongoing studies of clinical relevance.

Drug Type	Mechanism	Trial ID	Drugs Combination	Phase	Tumors	Status	Results
IL-2							
THOR-747	Non-alfa IL-2	NCT04009681	Anti-PD-1 (pembrolizumab), anti-EGFR (cetuximab)	1/2	Advanced malignancies	Active, not recruiting	ORR = 5.9%
MDNA11	Non-alfa IL-2	NCT05086692	Anti-PD-1 (pembrolizumab)	1/2	Advanced malignancies	Recruiting	ORR = 7.7%
ALKS-4230 (Nevmaleukin alfa)	Fusion protein (IL-2Ra)	NCT04592653	Anti-PD-1 (pembrolizumab)	1/2	Advanced malignancies	Active, not recruiting	n/a
AU007	Agonistic mAb	NCT05267626	Anti-PD-1 (avelumab), aldesleukin	1/2	Advanced malignancies	Recruiting	n/a
XTX202	IL-2 tumor-activated	NCT05052268	Single therapy	1/2	Advanced malignancies	Active, not recruiting	DCR = 31%
WTX-124	IL-2 prodrug	NCT05479812	Anti-PD-1 (pembrolizumab)	1	Advanced malignancies	Recruiting	Monotherapy: ORR = 30%
ODC-IL2	IL-2 prodrug	NCT06770764	Single therapy	1	Advanced malignancies	Recruiting	n/a
RO7284755 (Eciskafusp alfa)	Immunocytokine PD1-IL2v	NCT04303858	Anti-PD-L1 (atezolizumab)	1/2	Advanced malignancies	Recruiting	n/a
ANV600	Immunocytokine PD1-IL2v	NCT06470763	Anti-PD-1 (pembrolizumab)	1/2	Advanced malignancies	Recruiting	n/a
STAR0602 (invikafusp alfa)	Bifunctional Antibody-fusion (TCR/IL2)	NCT05592626	Single therapy	1/2	Advanced malignancies	Recruiting	DCR = 60%
IL-15							
N-803	IL-15 superagonist (intravesical)	NCT02138734	Intravesical BCG	1/2	NMIBC	Recruiting	n/a
	IL-15 superagonist (subcutaneous)	NCT06253494	Anti-PD-1 (pembrolizumab), lenvatinib, and HER2 Autologous DC vaccine	1/2	Endometrial cancer	Recruiting	n/a
	IL-15 superagonist (subcutaneous)	NCT06149481	SX-682, TriAdeno vaccine, and Retifanlimab	1/2	Advanced melanoma	Recruiting	n/a
SOT201	Immunocytokine PD-L1/IL-15	NCT06163391	Single therapy	1	Advanced malignancies	Recruiting	n/a
SAR445877	Immunocytokine PD-L1/IL-15	NCT05584670	Anti-EGFR (cetuximab)	1/2	Advanced malignancies	Recruiting	n/a

n/a = not available data (ongoing or recently completed trial); IL2v: interleukin-2 variant; DC: dendritic cell; NMIBC: non-muscle-invasive bladder cancer; ORR: overall response rate; DCR: disease control rate.

**Table 4:** Selected active trials involving TME-targeting therapies. This is a non-exhaustive, manually curated list intended to highlight ongoing studies of clinical relevance.

Drug Type	Mechanism	Trial ID	Drugs Combination	Phase	Tumors	Status	Results
Anti-CCR8							
CHS-114	Anti-CCR8 mAb	NCT06657144	anti-PD-1 (toripalimab)	1	Advanced malignancies	Not yet recruiting	n/a
		NCT05635643	single therapy	1	Advanced malignancies	Recruiting	n/a
LM-108	Anti-CCR8 mAb	NCT05199753	anti-PD-1	1/2	Advanced malignancies	Recruiting	Pooled analysis (gastric): ORR = 36.1%; G3/4 TRAEs = 37.5%
BAY 3375968	Anti-CCR8 mAb	NCT05537740	anti-PD-1 (pembrolizumab)	1	Advanced malignancies	Recruiting	n/a
S-531011	Anti-CCR8 mAb	NCT05101070	anti-PD-1 (pembrolizumab)	1/2	Advanced malignancies	Recruiting	n/a
GS-1811	Anti-CCR8 mAb	NCT05007782	anti-PD-1 (zimberelimab)	1	Advanced malignancies	Recruiting	n/a
BMS-986340	Anti-CCR8 mAb	NCT04895709	anti-PD-1 (nivolumab), chemotherapy	1/2	Advanced malignancies	Recruiting	n/a
AMG-355	Anti-CCR8 mAb	NCT06131398	anti-PD-1 (pembrolizumab)	1	Advanced malignancies	Recruiting	n/a
RO7502175	Anti-CCR8 mAb	NCT05581004	anti-PD-1 (pembrolizumab, anti-PD-L1 (atezolizumab)	1	Advanced malignancies	Recruiting	n/a
BGB-A3055	Anti-CCR8 mAb	NCT05935098	anti-PD-1 (tislellizumab)	1	Advanced malignancies	Recruiting	n/a
ABBV-514	Anti-CCR8 mAb	NCT05005403	anti-PD-1 (budigalimab)	1	Advanced malignancies	Recruiting	n/a
IPG7236	Anti-CCR8 mAb	NCT05142592	single therapy	1	Advances malignancies	Recruiting	n/a
QLP2117	Anti-CCR8 mAb	NCT05830045	single therapy	1	Advances malignancies	Recruiting	n/a
CM369	Anti-CCR8 mAb	NCT05690581	single therapy	1	Advances malignancies	Recruiting	n/a
HC006	Anti-CCR8 mAb	NCT06304571	single therapy	1	Advances malignancies	Recruiting	n/a
ZL-1218	Anti-CCR8 mAb	NCT05859464	anti-PD-1 (pembrolizumab)	1	Advances malignancies	Recruiting	n/a
Anti-CD47							
Evorpacept (ALX148)	Anti-CD47 fusion protein	NCT03013218	Anti-PD-1 (pembrolizumab), anti-HER2 (Trastuzumab), CT	1	Advanced solid tumors	Active, not recruiting	HNSCC ORR = 20%; NSCLC ORR = 5%; G/GEJ ORR = 21.1%
		NCT05524545	Enfortumab-vedotin	1	Advanced UC	Recruiting	ORR = 63%
HCB101	Anti-CD47 fusion protein	NCT05892718	Single therapy	1	Advanced solid tumors	Recruiting	
STI-6643	Anti-CD47 mAb	NCT04900519	Single therapy	1	Advanced solid tumors	Recruiting	n/a
IMC-002	Anti-CD47 mAb	NCT05276310	Single therapy	1	Advanced solid tumors	Recruiting	DCR = 45.5%
AUR103	Small molecule	NCT05607199	Single therapy	1	Advanced solid tumors	Recruiting	n/a
DS-1103a	Anti-SIRPα mAb	NCT05765851	Trastuzumab-deruxtecan	1	Advanced solid tumors	Recruiting	n/a
HMPL-A83	Anti-CD47 mAb	NCT05429008	Single therapy	1	Advanced solid tumors	Recruiting	n/a
TGF-β							
Galunisertib	Oral TGF-βR1 inhibitor	NCT05700656	CT	1/2	Advanced CRC	Recruiting	n/a
Vactovasertib	Oral TGF-βR1 inhibitor	NCT05588648	Single therapy	1/2	Osteosarcoma	Recruiting	n/a
		NCT03732274	Anti-PD-L1 (durvalumab)	1/2	Advanced NSCLC	Active, not recruiting	ORR = 30.8%
		NCT03724851	Anti-PD-1 (pembrolizumab)	1/2	CRC and G/GEJ adenocarcinoma	Active, not recruiting	ORR = 13.3%
LY3200882	Oral TGF-βR1 inhibitor	NCT02937272	CT, anti-PD-L1 (LY3300054)	1	Advanced malignancies	Active, not recruiting	Pancreatic: DCR = 75%
SRK-181	Anti-TGF-β1	NCT04291079	Anti-PD-1 or anti-PD-L1	1	Advanced malignancies	Active, not recruiting	ORR = 16.2%; DCR = 51.5%

n/a = not available data (ongoing or recently completed trial); mAb: monoclonal antibody; CT: chemotherapy; G/GEJ: gastric and gastroesophageal junction; HNSCC: head and neck squamous cell cancer; NSCLC: non-small cell lung cancer; CRC: colorectal cancer; UC: urothelial carcinoma; ORR: overall response rate; DCR: disease control rate; G3/4 TRAEs = grade 3–4 treatment-related adverse events.

**Table 5:** Selected active trials involving bispecific antibodies. This is a non-exhaustive, manually curated list intended to highlight ongoing studies of clinical relevance.

Drug Type	Mechanism	Trial ID	Drugs Combination	Phase	Tumors	Status	Results
T-cell engagers							
BA1202	CEA × CD3	NCT05909241	Single therapy	1	Advanced malignancies	Recruiting	n/a
TAK-186 (MVC-101	EGFR × CD3	NCT04844073	Single therapy	1/2	Advanced malignancies	Recruiting	n/a
CX-904	EGFR × CD3	NCT05387265	Single therapy	1	Advanced malignancies	Recruiting	n/a
KM257	HER2 × CD3	NCT05320874	Single therapy, CT	1	Advanced malignancies	Not yet recruiting	n/a
IMM2902	HER2 × CD3	NCT05805956	Single therapy	1/2	Advanced malignancies	Recruiting	n/a
Tarlatamab	DLL3 × CD3	NCT03319940	Single therapy	1	SCLC	Active, not recruiting	ORR = 23.4%;
Ubamatamab (REGN4018)	MUC16 × CD3	NCT03564340	Single therapy, anti-PD-1 (cemiplimab)	1/2	Ovarian cancer	Active, not recruiting	ORR = 18.2%
CC-1	PSMA × CD3	NCT04104607	Single therapy	1	CRPC	Recruiting	n/a
		NCT05646550	Single therapy	1	Prostate cancer	Recruiting	n/a
REGN4336	PSMA × CD3	NCT05125016	REGN5678 (PSMA × CD28), anti-PD-1 (cemiplimab)	1/2	CRPC	Recruiting	n/a
Xaluritamig (AMG-509)	STEAP1 × CD3	NCT04221542	Single therapy	1	CRPC	Recruiting	ORR = 22.7%
Cabotamig (ARB202)	CDH17 × CD3	NCT05411133	Single therapy	1/2	Advanced GI	Recruiting	n/a
XmAb819	ENNP3 × CD3	NCT05433142	Single therapy	1	Advanced RCC	Recruiting	n/a
JNJ-87890387	ENNP3 × CD3	NCT06178614	Single therapy	1	Advanced malignancies	Recruiting	n/a
EMB-07	ROR1 × CD3	NCT05607498	Single therapy	1	Advanced malignancies	Recruiting	n/a
BA1202	CEA × CD3	NCT05909241	Single therapy	1	Advances malignancies	Recruiting	n/a
AZD5863	Claudin18.2 × CD3	NCT06005493	Single therapy	1/2	Advanced GI	Recruiting	n/a
XmAb541	Claudin6 × CD3	NCT06276491	Single therapy	1	Advances malignancies	Recruiting	n/a
BNT-142	Claudin6 × CD3	NCT05262530	Single therapy	1	Advances malignancies	Recruiting	n/a
CC-3	B7-H3 × CD3	NCT05999396	Monotherapy	1	Advanced CRC	Recruiting	n/a
GEN1047	B7-H4 × CD3	NCT05180474	Monotherapy	1/2	Advanced solid tumors	Active, not recruiting	n/a
BA3182	CAB-EPCAM × CAB-CD3	NCT05808634	Single therapy	1/2	Advances adenocarcinoma	Recruiting	n/a
IM401 TCER^®^	MAGEA4/8 × TCR	NCT05359445	Single therapy, pembrolizumab	1	Advanced malignancies (restricted to HLA A*02:01)	Recruiting	DCR = 55%
IM402 TCER^®^	PRAME × TCR	NCT05958121	Single therapy	1/2	Advanced malignancies (restricted to HLA-A*02:01)	Recruiting	n/a
γδ T-cell engagers							
LAVA-1207	PSMA × CD3	NCT05369000	Single therapy, IL-2, pembrolizumab	1/2	CRPC	Active, not recruiting	n/a
NK-cell engagers							
AFM24	EGFR × CD16	NCT05109442	Anti-PD-L1 (atezolizumab)	1	Advanced malignancies	Recruiting	EGFR wt: ORR = 26.7%
Immunomodulators							
Dual inhibitory checkpoints							
IBI318	PD-1 × CTLA-4	NCT04777084	Single therapy	1	NSCLC	Recruiting	n/a
SSGJ-706	PD-1 × CTLA-4	NCT06533605	Single therapy	1	Advances malignancies	Not yet recruiting	n/a
Lorigerlimab (MDG019)	PD-1 × CTLA-4	NCT03761017	Single therapy	1	Advances malignancies	Active, not recruiting	CPRC cohort: ORR = 25.7%
Cadonilimab (AK104)	PD-1 × CTLA-4	NCT05426005	Single therapy	1	Advanced dMMR CRC	Recruiting	n/a
	PD-1 × CTLA-4	NCT05994001	LM-302 (Claudin18.2 × CD3)	1/2	Billiary tract cancer	Recruiting	ORR = 50%
SI-B003	PD-1 × CTLA-4	NCT04606472	Single therapy	1	Advances malignancies	Recruiting	ORR = 16.1%; DCR = 50%
Tobemstomig (RO7245669)	PD-1 × LAG-3	NCT04140500	Single therapy	1	Advances malignancies	Active, not recruiting	ORR = 17.1%; DCR = 51.4%
INCA32459	PD-1 × LAG-3	NCT05577182	Single therapy	1	Advances malignancies	Active, not recruiting	n/a
AK129	PD-1 × LAG-3	NCT05645276	Single therapy	1/2	Advances malignancies	Recruiting	n/a
AZD7789	PD-1 × TIM-3	NCT04931654	Single therapy	1/2	Advanced malignancies	Active, not recruiting	NSCLC: DCR = 47%; G3/4 TRAEs = 23%
LB1410	PD-1 × TIM-3	NCT05357651	Single therapy	1	Advanced malignancies	Recruiting	DCR = 35%
Rilvegostomig (AZD2936)	PD-1 × TIGIT	NCT04995523	Single therapy	1/2	Advanced NSCLC	Recruiting	ORR = 3,9%; DCR = 43%
BC008-1A	PD-1 × TIGIT	NCT06773481	Single therapy	1	Recurrent glioma	Not yet recruiting	n/a
HLX301	PD-L1 × TIGIT	NCT05102214	Single therapy	1	Advanced malignancies	Recruiting	n/a
Co-stimulatory bsAbs							
FS120	OX40 × 4-1BB	NCT05263180	Anti-PD-1 (pembrolizumab)	1	Advanced malignancies	Active, not recruiting	n/a
EMB-09	OX40 × PD-L1	NCT05263180	Single therapy	1	Advanced malignancies	Recruiting	n/a
XmAb^®^808	CD28 × B7-H3	NCT05585034	Pembrolizumab	1	Advanced malignancies	Recruiting	n/a
BNA035	4-1BB × EGFR	NCT05150457	Monotherapy	1	Advanced malignancies	Recruiting	n/a
ABL103	4-1BB × B7-H4	NCT06126666	Single therapy	1	Advanced malignancies	Recruiting	n/a
YH3267	4-1BB × HER2	NCT05523947	Monotherapy	1/2	Advanced malignancies	Recruiting	n/a
HLX35	4-1BB × EGFR	NCT05360381	Single therapy	1	Advanced malignancies	Active, not recruiting	n/a
PRS-344/S095012	4-1BB × PD-L1	NCT05159388	Single therapy	1	Advanced malignancies	Active, not recruiting	n/a
CB307	4-1BB × PSMA	NCT04839991	Single therapy	1	Advanced malignancies	Recruiting	n/a
ABL503	4-1BB × PD-L1	NCT04762641	Single therapy	1	Advanced malignancies	Recruiting	ORR = 15.3%; DCR = 61.5%
FS222	4-1BB × PD-L1	NCT05924906	Single therapy	1	Advanced malignancies	Recruiting	n/a
ALG.APV-527	4-1BB × 5T4	NCT05934539	Single therapy	1/2	Advanced malignancies	Recruiting	n/a
PM1032	4-1BB × Claudin18.2	NCT05839106	Single therapy	1/2	Advanced malignancies	Recruiting	ORR = 12.5%; DCR = 56.2%; G3/4 TRAEs: 10%
Anti-CD47 bsAbs							
Peluntamig (PT217)	DLL3 × CD47	NCT05652686	anti-PD-L1 (atezolizumab), CT	1/2	Neuroendocrine tumors/carcinoma	Recruiting	n/a
BAT7104	PD-L1 × CD47	NCT05767060	Single therapy	1	Advanced solid tumors	Recruiting	n/a
IMM2520	PD-L1 × CD47	NCT05780307	Single therapy	1	Advanced solid tumors	Recruiting	DCR = 54.5%; G3/4 TRAEs 56%
IMM2902	HER-2 × CD47	NCT05805956	Single therapy	1	Advanced solid tumors	Recruiting	DCR = 30.8%; G3/4 TRAEs 25%
IBC0966	PD-L1 × CD47	NCT04980690	Single therapy	1/2	Advanced solid tumors	Recruiting	n/a
HX009	PD-1 × CD47	NCT05731752	Single therapy	1	Advanced solid tumors	Active, not recruiting	n/a
TGF-β bsAbs							
YM101	PD-L1 × TGF-β	NCT05028556	Single therapy	1	Advanced malignancies	Active, not recruiting	n/a
BCA101	EGFR × TGF-β	NCT04429542	Anti-PD-1 (pembrolizumab)	1	Advances malignancies	Recruiting	ORR = 23.3%; DCR 81.8%

n/a = not available data (ongoing or recently completed trial); SCLC: small-cell lung cancer; CPRC: castration-resistant prostatic cancer; RCC: renal cell cancer; GI: gastrointestinal; dMMR: deficient mismatch repair; CRC: colorectal cancer; ORR: overall response rate; DCR: disease control rate; G3/4 TRAEs: grade 3–4 treatment-related adverse events.
